# Infection of Fungi and Bacteria in Brain Tissue From Elderly Persons and Patients With Alzheimer’s Disease

**DOI:** 10.3389/fnagi.2018.00159

**Published:** 2018-05-24

**Authors:** Ruth Alonso, Diana Pisa, Ana M. Fernández-Fernández, Luis Carrasco

**Affiliations:** Centro de Biología Molecular “Severo Ochoa”, Consejo Superior de Investigaciones Científicas y Universidad Autónoma de Madrid, Madrid, Spain

**Keywords:** polymicrobial infection, Alzheimer’s disease, infection in aging brain, fungal infection, next generation sequencing, bacteria and fungal co-infections

## Abstract

Alzheimer’s disease (AD) is the leading cause of dementia in elderly people. The etiology of this disease remains a matter of intensive research in many laboratories. We have advanced the idea that disseminated fungal infection contributes to the etiology of AD. Thus, we have demonstrated that fungal proteins and DNA are present in nervous tissue from AD patients. More recently, we have reported that bacterial infections can accompany these mycoses, suggesting that polymicrobial infections exist in AD brains. In the present study, we have examined fungal and bacterial infection in brain tissue from AD patients and control subjects by immunohistochemistry. In addition, we have documented the fungal and bacterial species in brain regions from AD patients and control subjects by next-generation sequencing (NGS). Our results from the analysis of ten AD patients reveal a variety of fungal and bacterial species, although some were more prominent than others. The fungal genera more prevalent in AD patients were *Alternaria*, *Botrytis*, *Candida*, and *Malassezia*. We also compared these genera with those found in elderly and younger subjects. One of the most prominent genera in control subjects was *Fusarium*. Principal component analysis clearly indicated that fungi from frontal cortex samples of AD brains clustered together and differed from those of equivalent control subjects. Regarding bacterial infection, the phylum *Proteobacteria* was the most prominent in both AD patients and controls, followed by *Firmicutes*, *Actinobacteria*, and *Bacteroides*. At the family level, *Burkholderiaceae* and *Staphylococcaceae* exhibited higher percentages in AD brains than in control brains. These findings could be of interest to guide targeted antimicrobial therapy for AD patients. Moreover, the variety of microbial species in each patient may constitute a basis for a better understanding of the evolution and severity of clinical symptoms in each patient.

## Introduction

Alzheimer’s disease (AD) is characterized by progressive memory impairment, with subsequent behavioral disturbances and profound deterioration of daily life activities (Blennow et al., 2006). AD is the leading cause of dementia in elderly people; accordingly, it is estimated that there are at present over 30 million patients with AD worldwide (Claeysen et al., 2012; Mayeux and Stern, 2012). The majority of AD cases are sporadic and only a subset (around 1–2%) has an early onset of the disease, which segregates with autosomal dominant mutations in three genes: β-amyloid precursor protein (*APP*), PSN1 and PSN2 (Newman et al., 2007; Guerreiro et al., 2012). In the late onset form of the disease, which is by far the most common, the best-established genetic risk factor is the association with the E4 allele of apolipoprotein E (*ApoE4*) (Saunders et al., 1993; Leoni, 2011; Liu et al., 2013). Additional risk factors include atherosclerosis, hypercholesterolemia, obesity and diabetes (Bagger et al., 2004; Barberger-Gateau et al., 2007; Mayeux and Stern, 2012; Shah, 2013; Vignini et al., 2013), although aging is considered the most important risk factor. Postmortem pathological features include the presence of extracellular deposits of amyloid-β (Aβ) plaques, intracellular neurofibrillary tangles of hyperphosphorylated tau protein, and neuronal loss (O’Brien and Wong, 2011; Revett et al., 2013). Aβ is a peptide of 39–42 amino acids that is generated by proteolytic processing of APP (O’Brien and Wong, 2011). Hyperphosphorylated tau protein polymerizes and is unable to interact with microtubules, leading to the generation of neurofibrillary tangles, which are damaging for cells (Himmelstein et al., 2012). The amyloid cascade hypothesis posits that the initial symptoms of the disease are caused by the deposition of Aβ that is produced by an imbalance between its production and clearance (Claeysen et al., 2012). The suggestion that Aβ increases tau phosphorylation, triggering cell death and AD, was hypothesized several years ago. This hypothesis, however, fails to explain several clinical symptoms of the disease, including systemic inflammation markers, and has been questioned by several researchers (Teich and Arancio, 2012).

Systemic inflammation is commonly observed in AD patients, including elevated levels of proinflammatory cytokines and also the presence of complement components in amyloid plaques (Sardi et al., 2011; Heneka et al., 2015; Heppner et al., 2015). Indeed, cerebrovascular lesions, including hemorrhages, microinfarcts and vascular degeneration, are observed in the vast majority of AD patients. These vascular disorders contribute to cognitive decline and the underlying pathology of the disease (Kalaria, 2002; Borenstein et al., 2005; Gorelick et al., 2011; Deramecourt et al., 2012).

We have provided extensive evidence that disseminated mycoses are implicated as causative agents or as risk factors for AD (Alonso et al., 2014a,b). Accordingly, we have demonstrated that fungal components can be found in post-mortem brain tissue from patients diagnosed with AD, and proteomic analyses revealed several peptides that unequivocally correspond to fungal proteins (Alonso et al., 2014a; Pisa et al., 2015b). Similarly, fungal DNA can be detected by nested PCR and DNA sequencing analysis, strengthening the evidence for the existence of a variety of fungal species in a single AD patient (Pisa et al., 2015b; Alonso et al., 2017a). Additionally, fungal macromolecules such as polyglucans, proteins and DNA can also be found in peripheral blood and cerebrospinal fluid from AD patients (Alonso et al., 2014b). More strikingly, we have directly observed yeast-like cells and hyphal structures in central nervous system (CNS) tissue from AD patients using specific polyclonal antibodies raised against a variety of fungi (Pisa et al., 2015a,b). These fungal structures were found associated with neural cells both intra- and extracellularly. Overall, these observations are consistent with the hypothesis that disseminated mycoses exist in AD patients.

Alzheimer’s disease and amyotrophic lateral sclerosis (ALS) share several clinical and neuropathological characteristics (Molteni and Rossetti, 2017), including the accumulation of amyloid protein (Takeda, 2017). In this regard, we have also found evidence of fungal structures and DNA in the cerebrospinal fluid of ALS patients (Alonso et al., 2015, 2017b). Further support for the concept that mycoses are present, even before the appearance of the disease, comes from the findings of elevated levels of chitinase detected in blood and cerebrospinal fluid from AD and ALS patients (Choi et al., 2011; Watabe-Rudolph et al., 2012; Rosen et al., 2014; Melah et al., 2015; Pagliardini et al., 2015). More recently, we have demonstrated the presence of bacterial infections and bacterial DNA in AD brain tissue (Pisa et al., 2017). Our analyses show that co-infections of fungi and bacteria can be detected using specific antibodies and DNA sequencing and, therefore, polymicrobial infections may represent the etiological factors in AD. Against this background, the finding that Aβ peptide possesses potent antifungal and antibacterial activities and is involved in the innate immune response against microbial infections (Soscia et al., 2010; Kumar et al., 2016) supports the notion that amyloid plaques represent an immune response to putative infections in AD patients. Of interest, similarities between amyloid oligomers and pore forming proteins have been noted, suggesting that both can increase membrane permeability, leading to cell death (Yoshiike et al., 2007).

In the present study, we have compared the fungal and bacterial infections in AD patients, elderly people and control subjects by immunohistochemistry. Additionally, we have endeavored to identify the different fungi and bacteria existing in brain tissue from AD patients using PCR and massive parallel sequencing, and have evaluated whether some of these species are common to all patients.

## Materials and Methods

### Description of AD Patients and Controls

Samples of brain sections and frozen tissue were obtained from patients diagnosed with AD or from control subjects. The age and gender of all subjects are listed in Supplementary Table S1. Most of the samples were supplied by the brain bank *Banco de Tejidos CIEN*, Madrid, and were analyzed anonymously. Some control samples of frozen tissue (control patients C14–C16) were obtained from the Netherlands Brain Bank. Sample transfer was carried out according to national regulations concerning research on human biological samples. The Ethics Committee of the Universidad Autónoma de Madrid approved the study. In all cases, written informed consent was obtained.

The majority of samples were processed according to a common postmortem protocol followed by *Banco de Tejidos CIEN*. Briefly, rapid neuropathological autopsy was performed upon call by the donor’s proxies (mean postmortem interval was 4.5 h). Immediately after extraction, the right half of the brain was sliced and frozen at -80°C, while the left half was fixed by immersion in phosphate-buffered 4% formaldehyde for at least 3 weeks. A full neuropathological study was performed on the left half brain after fixation. Neuropathological diagnosis and staging of all disease entities was performed according to consensus criteria. Various neuropathological variables related to AD, vascular, Lewy and TDP (TAR DNA-binding protein) pathologies, in addition to the presence of hippocampal sclerosis, were recorded for full classification of cases. Samples from the frozen tissue were obtained with sterile instruments in a laminar flow cabinet taking all measures to avoid contamination.

### Immunohistochemistry Analysis

CNS tissue was fixed in 10% buffered formalin for 24 h and embedded in paraffin following standard protocols. For immunohistochemical analysis, paraffin was removed and tissues were rehydrated and boiled for 2 min in citrate buffer and then incubated for 10 min with 50 mM ammonium chloride. Subsequently, tissue sections were incubated for 10 min with 0.1% Triton X-100 in phosphate buffered saline (PBS) and for 20 min with 2% bovine serum albumin (BSA) in PBS. Sections were incubated overnight at 4°C with primary antibodies in PBS/BSA. Thereafter, sections were washed with PBS and further incubated for 1 h at 37°C with the corresponding secondary antibody conjugated to Alexa 488 (Invitrogen, Carlsbad, CA, United States). Subsequently, tissue sections were stained with DAPI (Merck Millipore, Darmstadt, Germany) and samples were treated with autofluorescence eliminator reagent (Merck Millipore).

The following antibodies were used: rabbit polyclonal antibody against *Borrelia burgdorferi* (Genetex, Irvine, CA, United States), used at 1:50 dilution; rabbit polyclonal antibody against *Clostridium pneumoniae*, which immunoreacts with the major outer porin (Biorbyt, Cambridge, United Kingdom), used at 1:20 dilution; mouse monoclonal antibody against *Chlamydia* (Abcam, United Kingdom), used at 1:10 dilution; and mouse monoclonal antibody against peptidoglycan (Thermo Fisher Scientific, Waltham, MA, United States), used at 1:20 dilution. Rabbit polyclonal antibodies were as previously described (Pisa et al., 2015a). CNS tissue was embedded in paraffin following standard techniques and cut into 5 μm sections using a microtome (Microm HM355s; Microm, Walldorf, Germany). A previously described protocol was followed for immunohistochemical analysis. The majority of the images were collected on a Zeiss LSM710 multiphoton confocal laser scanning microscope equipped with the upright microscope stand AxioImager.M2 (Zeiss), and running ZEN 2010 software. Wide-fields were collected with the high-speed, high-resolution A1R+ confocal microscope (Nikon) combined with an inverted microscope, running NIS Elements 4.40 software. Images were deconvoluted using Huygens software (4.2.2 p0) and visualized with ImageJ (NIH).

### DNA Extraction From Frozen CNS Tissue

DNA was extracted from frozen samples of the following CNS regions: frontal cortex (FC), entorhinal cortex (ERH), medulla (MD), spinal cord (SC), superior frontal gire (GFS) and parietal cortex (PC) as described previously (Alonso et al., 2017a).

### Nested PCR

A number of measures were used to prevent PCR contamination including the use of separate rooms and glassware supplies for PCR set-up and products, aliquoted reagents, positive-displacement pipettes, aerosol-resistant tips and multiple negative controls. DNA samples from frozen CNS tissue were analyzed by nested PCR using several primer pairs. Primer design for amplification of the internal transcribed spacer (ITS) regions of fungal ribosomal DNA has been described in detail (Pisa et al., 2015b). The first PCR was carried out using 2 μl of DNA incubated at 95°C for 10 min, followed by 30 cycles of 45 s at 94°C, 1 min at 57.3°C and 45 s at 72°C. Oligonucleotides used in the first PCR were forward ITS-1 (external 1): ^1448^5′GTTCTGGGCCGCACGGG 3^′1465^ and reverse ITS-1 (external 1): ^106R^5′GGCAAAGATTCGATGATT3^′88R^. The second PCR was performed using 2 μl of the product obtained in the first PCR and ITS-1 (internal 1A) primers for 35 cycles of 45 s at 94°C, 1 min at 55°C and 45 s at 72°C. The oligonucleotides used were forward ITS-1 (internal 1A) ^1771^5′TCCGTAGGTGAACCTGCGG3^′1790^ and reverse ITS-1 (internal 1A) ^50R^5′GCTGCGTTCTTCATCGATGC3^′30R^. We also used internal 1B primers for 30 cycles of 45 s at 94°, 1 min at 52° and 45 s at 72°. The oligonucleotides used were forward ITS-1 (internal 1B) 5′GCGTCTA GACCTGCGGAAGGATCA 3′ and reverse (internal 1B) 5′GCGAAGCTTGATCCGTTGTTGAAA3′. A separate PCR assay was designed to amplify the ITS-2 region. The first PCR assay was carried out with 2 μl of DNA incubated at 95°C for 10 min, followed by 30 cycles of 45 s at 94°C, 1 min at 52°C and 45 s at 72°C. Oligonucleotides used in the first PCR were forward ITS-2 (External 2)^152^5′TTTCAACAACGGATCTC3^′169^ and reverse ITS-2 (External2) ^858^5′AGTACGGGATTCTCACCCTC3^′838^. The second PCR was carried out with 2 μl of the product obtained in the first PCR and ITS-2 (internal 2) primers for 30 cycles of 45 s at 94°C, 1 min at 55°C, and 45 s at 72°C. Oligonucleotides used were forward ITS-2 (internal2′)^274^5′GCATCGATGAAGAACGCAGC3^′295^ and reverse ITS-2 (internal 2):^572R^5′TCCTCCGCTTATTGATATGC3^′552R^.

The human β-globin gene served as a control for DNA extraction. PCR was carried out with 4 μl of DNA incubated at 95°C for 10 min and amplified with 42 cycles of 45 s at 94°C, 1 min at 60°C and 45 s at 72°C. The oligonucleotides used were 5′GGTTGGCCAATCTACTCCCAGG3′and 5′GCTCACTCAGTGTGGCAAAG3′. Amplified DNA products were analyzed by agarose gel electrophoresis and stained with ethidium bromide. PCR products were sequenced by Macrogen (Seoul, South Korea). The sequences have been deposited in the European Nucleotide Archive (ENA^1^).

### Next-Generation Sequencing

#### Fungi

The yeast ITS-1 region is highly variable both in length and in nucleotide sequence, and for this reason, it has utility in metagenomic next-generation sequencing (NGS) studies. The region between the internal 1 primers was amplified with specific primers joined to linker sequences in a first round of PCR (specific product of ∼300 nt). A second PCR was performed on this product using fusion primers containing Illumina and linker sequences.

#### Bacteria

Primers were designed to amplify the region between V3–V4 of 16S rDNA gene. These primers were joined to linker sequences in a first round of PCR (specific product of ∼400 nt). A second PCR was performed on this product using fusion primers containing Illumina and linker sequences.

The PCR products were sequenced on a MiSeq sequencing platform (Illumina). PCR and sequencing were performed by the Genomics Unit at the Scientific Park of Madrid. Quality analyses were performed over reads using FastQC software^2^. All sequences have been submitted to European Genome-phenome Archive with the accession number EGAS00001002766.

### Computational Analysis

#### Qiime Analysis

We used QIIME software for metagenomic analysis of fungi and bacteria (Caporaso et al., 2010). This is an open-source bioinformatics pipeline for performing microbiome analysis from raw DNA sequencing data. QIIME is designed to take users from raw sequencing data generated on the Illumina or other platforms to publication-quality graphics and statistics. This includes demultiplexing and quality filtering, operational taxonomic unit (OTU) picking, taxonomic assignment and phylogenetic reconstruction, and diversity analyses and visualizations. The adapters from the sequences were deleted using Cutadapt and all sequences with a length shorter than 35 bp were discarded. Once sequence set-up was ready, we performed a metagenomic-type analysis that consisted of several steps^3^. As a reference, we used the most recent version of the Qiime Fungal ITS database^4^.

#### Sequence Clustering

The sequences of all samples were grouped to define the OTUs using the pick_open_reference_otus.py workflow^5^ with a percentage identity of 97 and 95% in fungi and bacteria, respectively.

#### Principal Component Analysis

The Bray–Curtis distance matrix and the weight score of each principal component was calculated using the QIIME script core diversity analyses.py. The three-dimensional plot model of the principal component analysis (PCA) was done with the scatterplot3d package in R.

#### Identification of Uncultured Fungus Hits OTUs

According to the taxonomical classification, we found that on average 58% of the matches corresponded to “Uncultured fungus Blast” hit. For this reason an additional standard Blast search analysis was performed.

## Results

### Immunohistochemistry Analysis of Fungal Infection in AD Patients and in Elderly and Younger Controls

**FIGURE 1:**
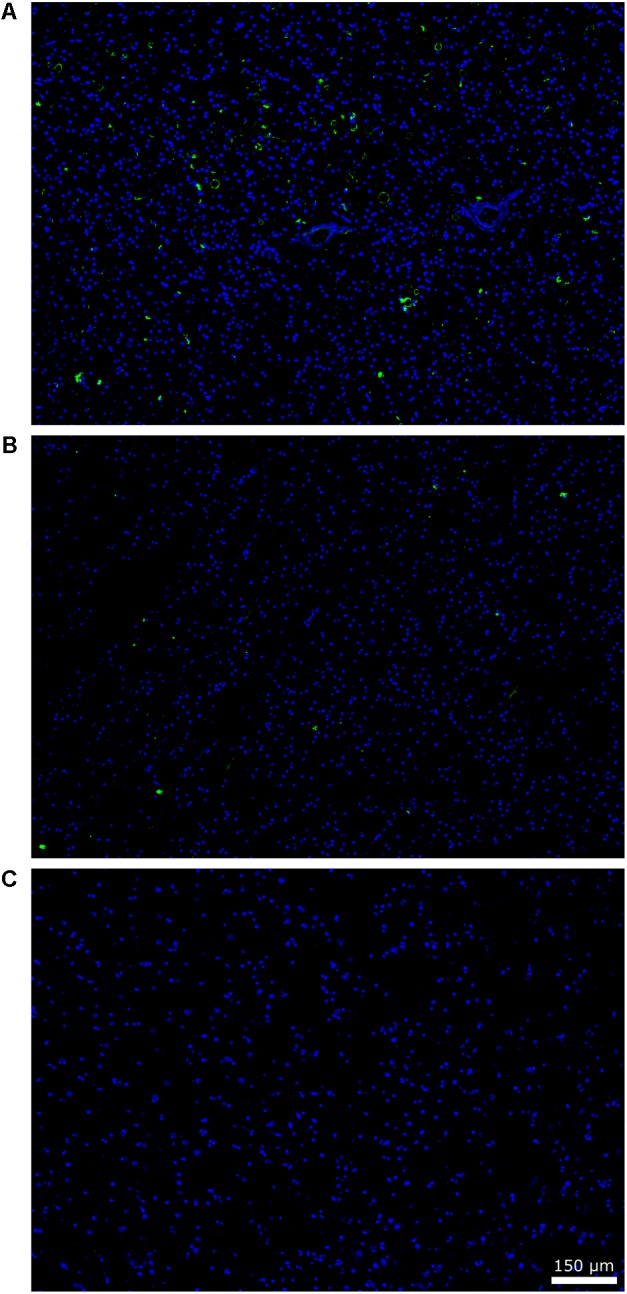
Immunohistochemistry of fungal structures brain sections. Paraffin-embedded brain sections (5 μm) were processed as described (see section “Materials and Methods”). Sections were incubated with rabbit polyclonal anti-*C. albicans* antibodies (1:100) and an Alexa 488-conjugated donkey anti-rabbit antibody (1:500) (green) and stained with DAPI (blue). A wide file (20× magnification) is shown for the ERH brain section of patient AD11 **(A)**, an elderly subject (C13) **(B)**, and a younger subject (C10) **(C)**. Scale bar is shown in the figure.

The aim of the study was to gain further insight into polymicrobial infections in brain tissue from AD patients, and to compare these findings with those from elderly people and younger subjects. Our previous results showed that tissue sections from different CNS regions of AD patients contain not only a number of yeast-like cells and hyphal structures (Pisa et al., 2015a,b), but also prokaryotic-like cells (Pisa et al., 2017). We first performed immunohistochemistry analysis for *C. albicans* on brain tissue from one AD patient (aged 83), one control subject (aged 53), and one elderly individual (aged 83) without degenerative diseases. **Figure 1** shows wide-field images of ERH sections from the three subjects. Various mycotic structures immunoreacting with the *C. albicans* antibody were detected in the ERH tissue of AD patient AD11 (**Figure 1A**), whereas a limited number of fungal structures could be observed in the elderly subject (C13; **Figure 1B**). By contrast, ERH tissue from the younger subject (C10) was practically devoid of immunoreactive material (**Figure 1C**). The rabbit polyclonal anti-*C. albicans* antibody cross-reacts with a variety of fungal species, and thus also reveals the presence of fungi that may not necessarily correspond to *C. albicans*.

We extended this analysis to CNS sections from other AD patients, elderly and younger subjects, using the same antibody. The numbers of immunopositive structures that showed a yeast-like or hyphal morphology were counted in all the sections examined and normalized by estimating the area analyzed. The median of the fungal structures per cm^2^ found was 14.01 ± 7.65 in sections from five AD patients (medium age 83.2 years old), 6.70 ± 3.17 in sections from five elderly subjects (medium age 79.4 years old), and 3.54 ± 1.63 in sections from five younger persons (medium age 49.8 years old). These findings indicate that the number of fungal structures in brain sections was lower in elderly subjects than in AD patients, and even lower in younger controls. A Kruskal–Wallis *H* test was done to corroborate significant difference and found *p* = 0.0070. The Pairwise Wilcoxon test was then used in order to calculate pairwise comparisons between the different groups with corrections for multiple testing. The AD group showed significant difference when compared to the elderly subjects (*p* = 0.024) and younger subjects (*p* = 0.024). Comparing elder and younger groups, lower difference was found (*p* = 0.151). In conclusion, while mycotic structures can be found in brain tissue from elderly subjects, the burden of this infection is higher in AD patients.

### Nested PCR Analysis of Fungal Infection in CNS Tissue of AD Patients

**FIGURE 2:**
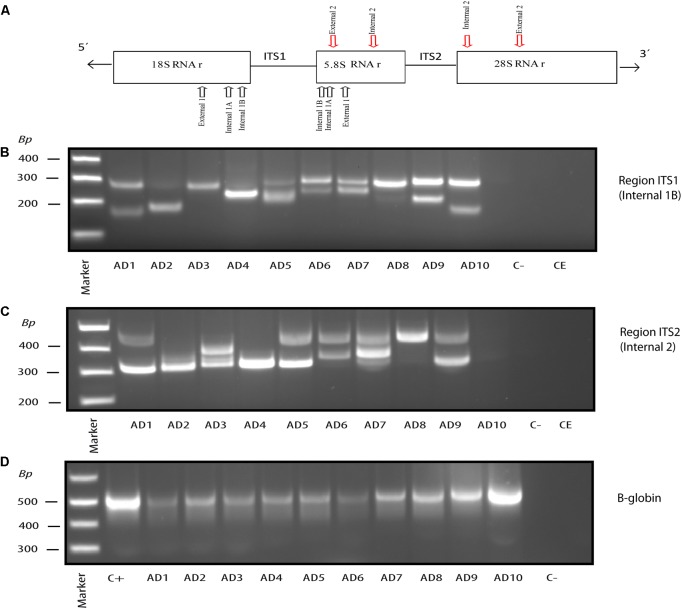
Nested PCR of fungal DNA from AD frozen tissue. PCR analysis was carried out as described (see section “Materials and Methods”). Schematic representation of fungal rRNA genes (18S, 5.8S and 28S rRNA) and the ITS-1 and ITS-2 regions, including location of the primers employed for the different nested PCRs: primers External 1 employed in the first PCR; primers Internal 1A and Internal 1B employed in the second PCR to amplify ITS-1; primers Internal 2 employed in the second PCR to amplify ITS-2 **(A)**. Agarose gel electrophoresis of the DNA fragments amplified by nested PCR using DNA extracted from frozen FC tissue. PCR analysis of ten AD patients using primers Internal 1B to amplify the ITS-1 region **(B)**. PCR analysis to amplify the ITS-2 region from 10 AD patients using primers Internal 2 **(C)**. PCR analysis of DNA extracted from the samples tested in **(B,C)** using human β-globin oligonucleotide primers **(D)**. Control–, PCR without DNA. CE, Control of DNA extraction without DNA. C+, DNA extracted from HeLa cells.

We have previously demonstrated the presence of DNA from a variety of fungal species in frozen tissue from the ERH region of several AD patients (Alonso et al., 2017a). To do this, we employed nested PCR analyses of the ITS-1 and ITS-2 regions, which are intergenic sequences located between the rRNA genes (see scheme **Figure 2A**). In our experience, the use of different primers to amplify these sequences can render different fungal species, and so it is important to amplify both regions to survey as many fungal species as possible. We used this approach to interrogate DNA from frozen tissue of the FC from ten AD patients. **Figures 2B,C** show the fragments obtained in each patient from ITS-1 and ITS-2 regions, respectively. As a control for DNA extraction and to demonstrate the presence of DNA in each sample, we also used PCR to amplify the human β-globin gene (**Figure 2D**). In addition, controls to demonstrate the absence of contamination in the PCR assay and the DNA extraction protocols are also shown. As observed previously from the ERH region, a number of different DNA fragments were found using FC samples. All fragments were extracted from agarose gels and sequenced. The fungal species are listed in **Table 1** and included those belonging to the genera *Alternaria*, *Cladosporium*, *Cryptococcus*, *Fusarium*, and *Malassezia*. Of interest, some of the species detected in the FC region were common to those found in the ERH region of AD patients, including *Cladosporium*, *Cryptococcus* and *Malassezia*.

**Table 1:** Fungal species detected in the frontal cortex (FC) of Alzheimer’s disease (AD) patients by PCR and DNA sequencing.

Species	Region ITS1	Region ITS2
*Alternaria Alternata*		AD5
*Cladosporium* sp.	AD2, AD7	AD1
*Cryptococcus magnum*	AD1	
*Cryptococcus* sp.	AD10	
*Fusarium merismoides*		AD3, AD4, AD9
*Malassezia globosa*	AD3, AD8	
*Malassezia restricta*	AD10	AD5, AD6, AD9
*Sporobolomyces* sp.	AD10	
*Uncultured aureobasidium*	AD9	
*Uncultured fungus*	AD4, AD5	
*Uncultured malassezia*	AD1	AD3, AD8


### NGS Analysis of Fungal Infection in CNS Tissue of AD Patients

**FIGURE 3:**
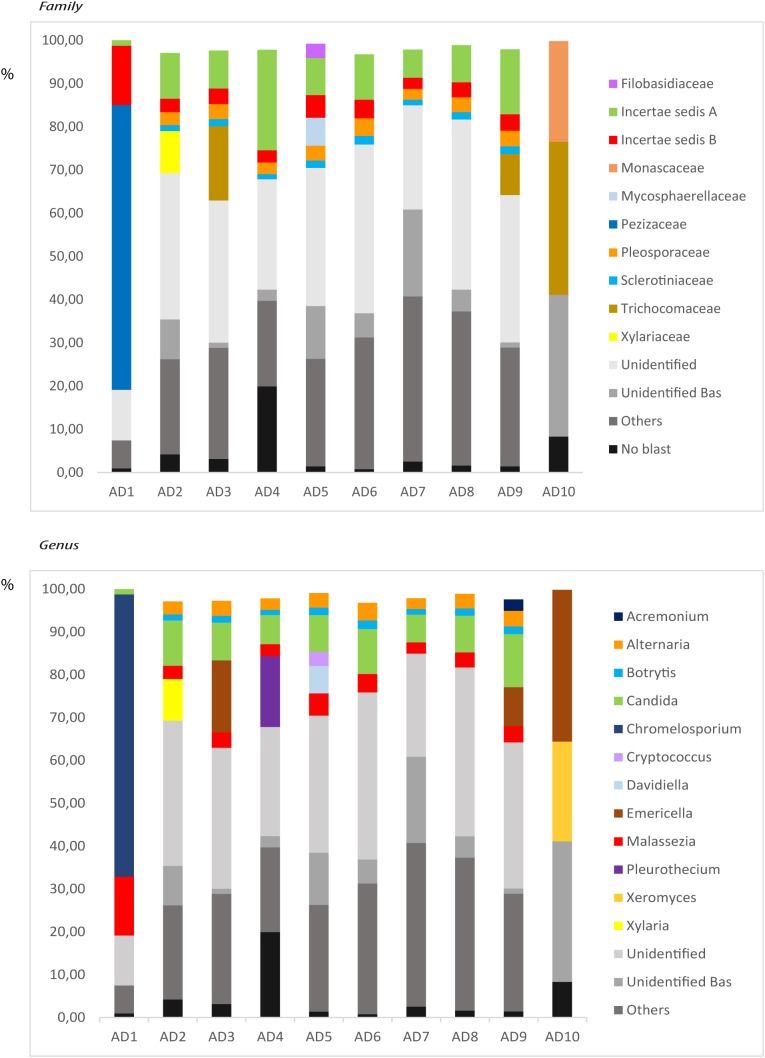
Distribution of fungal families and genera obtained by NGS of DNA from ten AD patients. Computational analyses of the sequences obtained on the Illumina platform using Qiime classified the data into fungal families and genera. **(Upper)** Shows the results of fungal families obtained from FC of AD patients. **(Lower)** Shows the results of fungal genera obtained from FC of AD patients. Asc, *Ascomycota*; Bas, *Basidiomycota*; Chy, *Chytridiomycota*.

For a more in-depth comparison of the different fungal species present in the brain regions of AD patients, we next performed NGS. Accordingly, extracted DNA from frozen FC tissue of the ten AD patients analyzed by nested PCR was sequenced using the Illumina platform. About 209,000–360,000 sequences were obtained for each sample and the results were processed by bioinformatic tests, as detailed in Section “Materials and Methods.” A great variety of fungal species was apparent in FC tissue from each patient. The species with an abundance >1% in each patient are listed in Supplementary Table S2, and fungal families and genera present are indicated in **Figure 3**. As previously observed for the ERH region, there was a variety of species detected in each AD patient and they varied from one patient to another. The most prevalent genera in the 10 patients were *Alternaria*, *Botrytis*, *Candida*, and *Malassezia*. In addition, other genera such as *Chromelosporium, Cryptococcus, Davidiella*, and *Emericella* were also found in significant percentages. A comparison of the fungal species and genera obtained by PCR and NGS, including those species detected below 1%, are listed in Supplementary Table S3. Finally, the comparison of the genera obtained by NGS in our previous work using the ERH region of eight AD patients (Alonso et al., 2017a) with the results obtained in this work analyzing the FC tissue, is shown in Supplementary Table S4. The most prevalent genera common to both ERH and FC of AD patients included *Alternaria, Botrytis, Candida*, and *Malassezia.* In conclusion, the majority of the fungal genera found in this work are coincident with those identified in our previous works (Pisa et al., 2015b; Alonso et al., 2017a) and thus appear to be common to both regions.

### Nested PCR Analysis of Fungal DNA in CNS Tissue From Control Subjects

**FIGURE 4:**
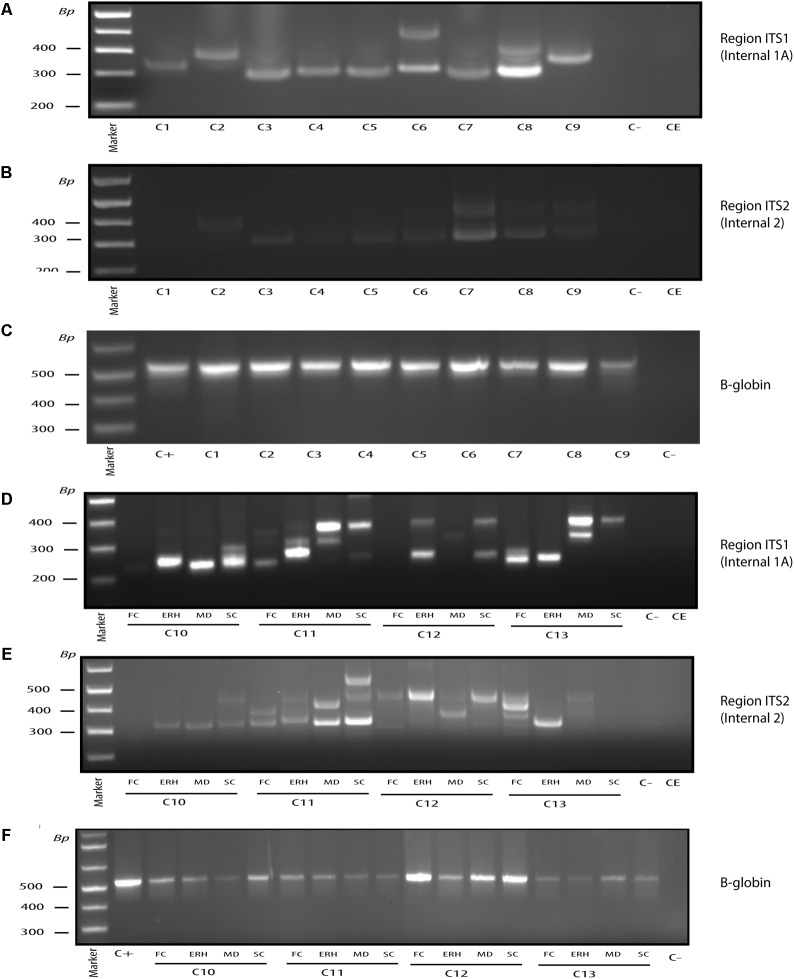
Nested PCR of fungal DNA from control individuals. PCR analysis was carried out as described (see section “Materials and Methods”). Agarose gel electrophoresis of the DNA fragments amplified by nested PCR. PCR analysis of DNA extracted from frozen ERH tissue of nine controls using primers Internal 1A to amplify the ITS-1 region **(A)**. PCR analysis to amplify region ITS-2 from ERH of nine controls using primers Internal 2 **(B)**. PCR analysis of DNA extracted from the samples tested in **(A,B)** using human β-globin oligonucleotide primers **(C)**. PCR analysis from FC, ERH, MD, and SC of four controls using primers Internal 1A to amplify the ITS-1 region **(D)**. PCR analysis to amplify ITS-2 region of the same control samples indicated in **(D)** using primers Internal 2 **(E)**. PCR analysis of DNA extracted from the samples tested in **(D,E)** using human β-globin oligonucleotide primers **(F)**. Control–, PCR without DNA. CE, control of DNA extraction without DNA. C+, DNA extracted from HeLa cells.

An emerging concept from recent observations is that human internal tissues that should be “sterile” actually contain a diversity of bacterial species, as revealed by the identification of bacterial DNA (Padgett et al., 2005; Marques da Silva et al., 2006; Ott et al., 2006; Koren et al., 2011; Leech and Bartold, 2015). Moreover, bacteria can be found in nervous tissue from normal subjects (Branton et al., 2016; Emery et al., 2017). To our knowledge, the possibility that fungi can be found in brain tissue in the control population has not been studied. We therefore tested whether fungal infection could be found in control CNS samples. In previous works, we examined only a limited number of controls, which were practically devoid of fungal DNA as tested by PCR, whereas NGS analysis of two samples of CNS from one control subject rendered a variety of fungal species (Alonso et al., 2014a, 2017a). As before, we first performed nested PCR of ITS-1 and ITS-2 regions in ERH brain tissue from nine control subjects (**Figures 4A,B**). Notably, a small number of amplified DNA fragments were detected in most of the subjects, with *Fusarium* being the most prevalent genus in control samples. To further test for fungal DNA in the CNS, three additional regions from four control subjects were tested: FC, MD, and SC. The DNA fragments amplified by nested PCR of ITS-1 and ITS-2, respectively, are shown in **Figures 4D,E**. DNA fragments were sequenced and the fungi are listed in **Table 2**. Consistent with previous results, when found, the DNA fragment amplified varied depending on the control subject and the CNS region examined. No DNA fragments were amplified in some CNS areas of controls C10 FC, C12 FC, C12 MD, and C13 SC, suggesting that fungal abundance is lower than that of AD patients (Alonso et al., 2017a). These findings also indicate that no fungal contamination occurs during sample preparation. This conclusion is reinforced by the fact that no DNA was amplified in the controls employed for DNA extraction and PCR, whereas PCR of the human β-globin gene was positive in all the DNA samples analyzed (**Figures 4C,F**).

**Table 2:** Fungal species detected in different brain regions of control subjects by PCR.

Region ITS1		Region ITS2	
**Species**	**Controls**	**Species**	**Controls**
*Candida albicans*	C7	*Candida albicans*	C7
*Candida glabrata*	C11SC	*Cladosporium* sp.	C11LF, C11SC
*Cladosporium* sp.	C12ERH,	*Fusarium oxysporum*	C3, C8, C10MD
*Fungal* sp.	C6	*Malassezia restricta*	C8, C12ERH
*Fusarium oxysporum*	C3, C4, C5, C8, C10MD	*Penicillium crustosum*	C11ERH
*Malassezia globosa*	C13MD	*Phoma* sp.	C13ERH
*Malassezia restricta*	C11SD, C12SC	*Saccharomyces cerevisiae*	C2
*Penicillium crustosum*	C11ERH	*Uncultured eukaryote*	C7
*Phoma* sp.	C13ERH	*Uncultured fungus*	C11SC
*Rhodotorula mucilaginosa*	C12SC	*Uncultured malassezia*	C11MD, C11SC, C12SC, C13LF
*Uncultured fungus*	C1, C10ERH, C11SC, C13LFC, C13MD	*Pichia membranifaciens*	C11MD
*Uncultured malassezia*	C11MD, C13SC		
*Uncultured rhodotorula*	C11LF		


**FC, frontal cortex; ERH, entorhinal cortex; MD, medulla; SC, spinal cord.**

### NGS Analysis of Fungal Infection in CNS Tissue From Control Subjects

**FIGURE 5:**
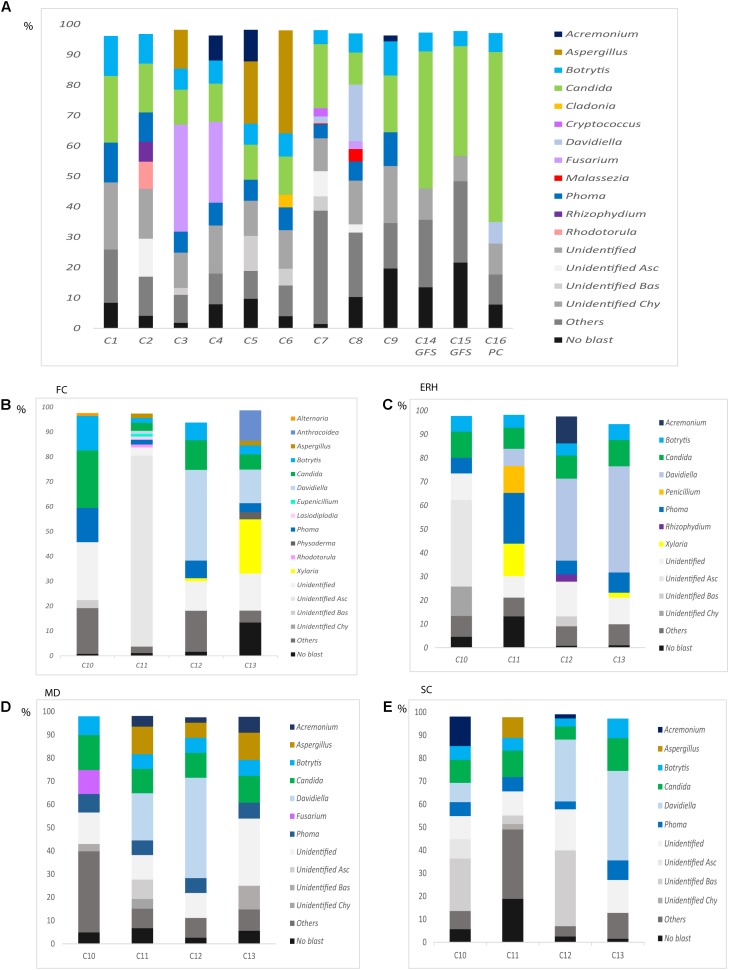
Distribution of fungal genera obtained by NGS of DNA from control subjects. Computational analyses of the sequences obtained on the Illumina platform using Qiime classified the data into fungal genera. Results of fungal genera obtained from DNA extracted from nine samples of frozen ERH tissue of controls (C1–C9) and two Superior Frontal Gire controls (C14, C15) and one Parietal cortex control (C16) **(A)** Results of fungal genera obtained from four CNS regions: FC, ERH, MD, and SC from four controls **(B–E)**. Asc, *Ascomycota*; Bas, *Basidiomycota*; Chy, *Chytridiomycota*.

We next used NGS in an attempt to gain a better understanding of the human brain mycobiome (i.e., the fungal genomes and hence the fungal biota). This was done using DNA extracted from frozen samples of 12 control subjects and from the four regions of four AD patients described above. Using the Illumina platform, about 204,000–336,000 sequences were obtained for each sample and the results were processed by bioinformatic tests. Initially, 12 DNA samples from the human brain cortex obtained from control individuals with different ages were used. The fungal species detected in each sample >1% are listed in Supplementary Table S5. Moreover, the fungal genera found in brain tissue from each control subject is shown in **Figure 5A**. Interestingly, *Fusarium*, which was the most prominent genus found by nested PCR, was also found in the same control subjects by NGS. In addition, *Aspergillus*, *Botrytis*, *Candida*, *Phoma*, *Malassezia*, among others were also found using this technique.

We also examined by NGS the four different CNS regions (FC, MD, ERH, and SC) from four controls, as indicated above using nested PCR. The fungal species determined in these samples are shown in Supplementary Table S6, and the fungal genera are depicted in **Figures 5B–E**. Of note, a variety of fungal genera were found in each region. For example, *Aspergillus* was detected in the CNS regions FC, MD, and SC from C11, and in the MD from C12 and C13. *Davidiella* was detected in the FC, ERH, MD, and SC regions from C11 and C12, and in the FC, ERH, and SC regions from C13. Some of these genera were common to the four brain areas analyzed, for instance *Botrytis*, *Candida*, and *Phoma*, while others were found only in one or two of these CNS regions, for instance, *Alternaria*, (C10 FC), *Fusarium* (C10 MD), and *Rhodotorula* (C11 FC).

### Comparison of Fungal Infection Between AD Patients and Control Subjects

**FIGURE 6:**
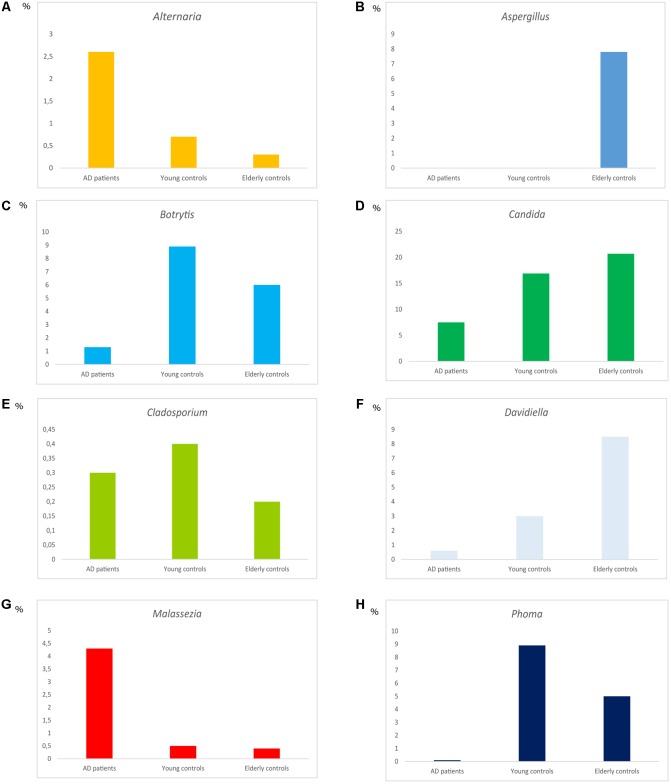
Distribution of fungal genera between AD patients, elderly and younger controls. Median of the percentages of the different genera as indicated in the Figure **(A–H)**.

One of the aims of the present work was to compare the fungal genera detected in CNS tissue in AD with that found in elderly and younger individuals. The most relevant genera and their percentages in brains from the three groups is shown in **Figure 6**. The percentage of some of these genera, such as *Alternaria* and *Malassezia*, was higher in AD patients than in control samples (**Figures 6A,G**). Also, *Aspergillus*, *Candida*, and *Davidiella* were found in a higher percentage in elderly than in AD patients and younger subjects (**Figures 6B,D,F**). Curiously, *Botrytis* and *Phoma* exhibited higher percentages in younger persons (**Figures 6C,H**). Finally, the percentage of *Cladosporium* was similar between the three groups examined (**Figure 6E**). Because a comparison of fungal burden present in brain tissue from different individuals cannot be achieved by NGS, these results reflect the median of the percentages, but not the total amount of these genera. The burden of fungal infection would be expected to be higher in AD patients than in elderly and younger individuals. Therefore, while the percentage of *Cladosporium* is similar between AD and controls, the actual abundance of this fungus should be higher in AD.

**FIGURE 7:**
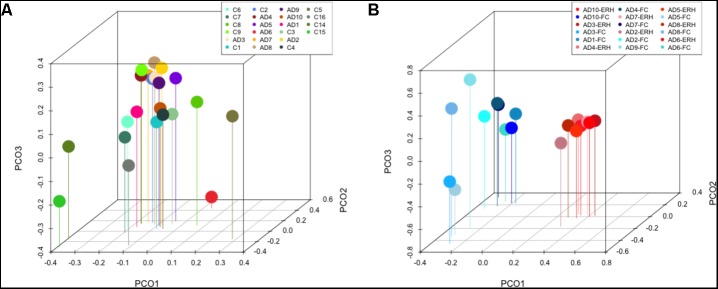
Principal component analysis of AD and control samples: 3D principal component analysis scatter plots of AD patients and controls. Distribution between ten AD patients using FC samples, nine control ERH samples, two Superior Frontal Gire control samples (C14 and C15), and one Parietal cortex control sample (C16) **(A)**. Distribution between ten AD patients using FC samples (plots in blue) and nine ERH samples (plots in red) **(B)**. The UniFrac method was used to calculate this parameter.

Principal component analysis of AD patients and controls is shown in **Figure 7A**. Notably, most AD patients were clustered together, suggesting a relationship between them, although AD6, who is the oldest patient, was not in the clustered group. By contrast, the control subjects were more scattered, indicating a higher diversity between them; for example, control C2 and C8 are closer to AD patients. Of interest, samples from control subjects C14, C15, and C16 were clustered together and apart from the other controls. These three samples were supplied by Netherlands brain bank, and perhaps their mycobiome reflects the different geographical origin of the controls. Remarkably, comparison of PCA results of FC samples with those from the ERH region recently published by our group (Alonso et al., 2017a), all from AD patients, revealed that both FC and ERH are located in different regions, suggesting that the diversity of fungal species are similar in each group (**Figure 7B**). Thus, FC and ERH can be differentiated by this analysis, pointing once again that these fungal species do not result as a consequence of contamination during sample preparation.

### Analysis of Bacterial Co-infection in AD, Elderly Persons and Younger Subjects

**FIGURE 8:**
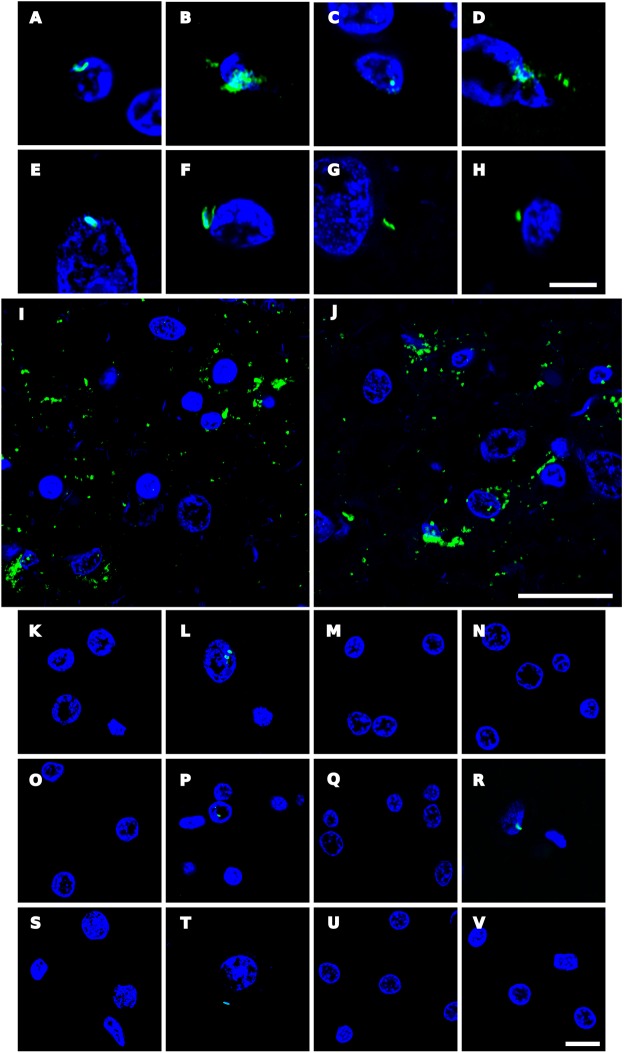
Immunohistochemistry analysis of bacterial structures in brain sections. Paraffin-embedded ERH sections (5 μm) were processed as indicated (see section “Materials and Methods”) and incubated with mouse monoclonal antibody against peptidoglycan at 1:20 dilution **(A–D**, **K–M,S)**. Sections were incubated with rabbit polyclonal antibody against *C. pneumoniae* used at 1:20 dilution **(E,F,N–P,T)**. Sections were incubated with mouse monoclonal antibody against *Chlamydia* used at 1:10 dilution **(G,H,Q,U)**. Sections were incubated with rabbit polyclonal antibody against *Borrelia burgdorferi* used at 1:50 dilution **(I,J,R,V)**. In all cases, DAPI staining appears in blue. **(A)** Patient AD2; **(B,C,F)** patient AD4; **(D)** patient AD5; **(E)** patient AD3; **(G,I,J)** patient AD7; **(H)** patient AD10; **(K,N)** control C11; **(L,O)** control C12; **(M,P)** control C13; **(Q,R)** control C6; **(S,T)**: control C10; **(U)** control C2, and **(V)** control C1. **(A–J,P–R,T–V)** ERH samples; **(K)** MD sample; **(L,N,S)** FC samples; **(M,O)** SC samples. Scale bar: 5 μm for **(A–H)**; 20 μm for **(I,J)**; and 10 μm for **(K–V)**.

We recently reported prokaryotic structures in AD brains that could be immunolabelled with several anti-bacterial antibodies (Pisa et al., 2017). Similarly, we found here that some prokaryotic-like cells and disorganized material could be detected in AD brain tissue by immunostaining with anti-peptidoglycan, anti-*Clamidophyla* or anti-*Borrelia* antibodies (**Figure 8**). Interestingly, some of these cells were found intranuclearly (**Figures 8A–E,I,J**), whereas on other occasions scattered material, without a specific morphology, was observed (**Figures 8B,D**). Moreover, in some instances, such as those observed with anti-*Borrelia* antibodies, some areas with numerous prokaryotic-like immunopositive cells were observed, suggesting that there are foci of bacterial infections (**Figures 8I,J**). By contrast, the burden of these structures revealed by anti-bacterial antibodies was lower in brain sections from elderly subjects and only very seldom were found in younger persons (**Figure 8**). In conclusion, immunohistochemistry studies employing anti-bacterial antibodies may serve to detect prokaryotic structures, as well as to compare their burden in AD and control individuals. These observations are in good agreement with the recent finding that lipopolysaccharide and *Escherichia coli* were detected by immunohistochemistry in brain parenchyma and vessels in all AD and control brains, although these levels were higher in AD patients (Zhan et al., 2016).

### NGS Analysis of Bacterial Infection in CNS Tissue From AD Patients

**FIGURE 9:**
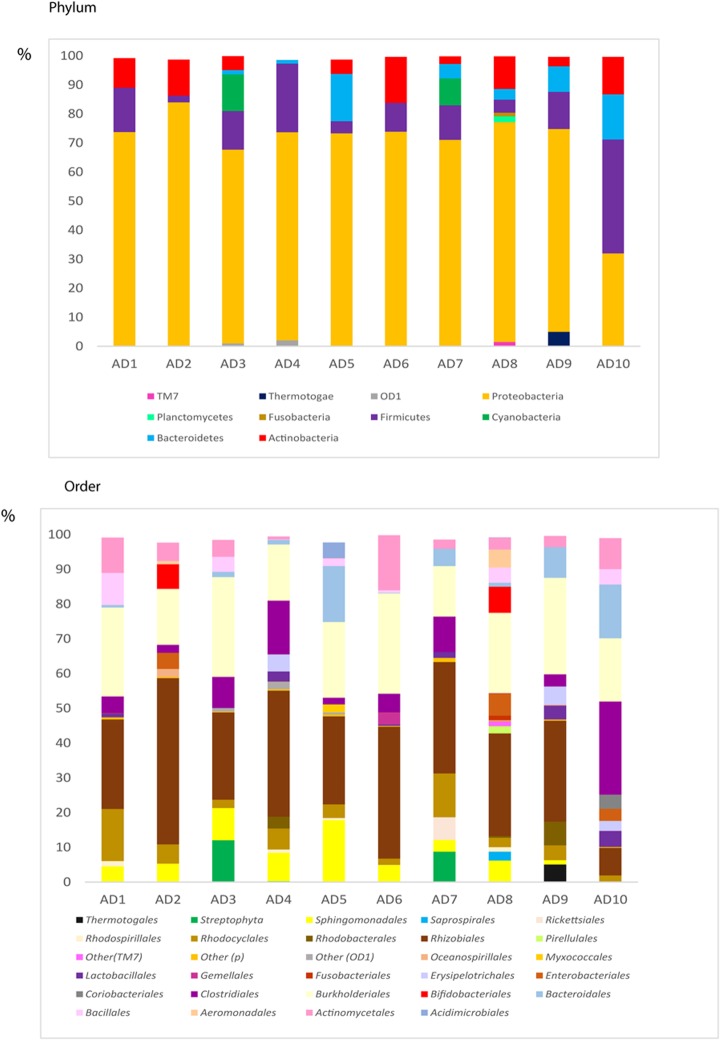
Distribution of bacteria phyla and orders obtained by NGS of DNA from AD patients. Computational analyses of the sequences obtained on the Illumina platform using Qiime classified the data into bacteria phyla and orders. **(Upper)** Shows the results of bacteria phyla obtained from ERH samples from 10 AD patients. **(Lower)** Shows the results of bacterial orders obtained from ERH samples from 10 AD patients.

We recently demonstrated that AD brain ERH tissue also contains bacterial DNA, as shown by nested PCR of the V3–V4 region of prokaryotic 16S rRNA gene (Pisa et al., 2017). These findings were consistent with those published simultaneously by another group, which also provided evidence of bacterial DNA in brain tissue from AD patients using NGS (Emery et al., 2017). To further characterize the bacterial communities in our samples, we used NGS to survey the bacterial sequences in 10 AD patients using the same V3–V4 region PCR samples, which revealed a great variety of bacterial species (Supplementary Table S7). The bacterial phyla and orders are shown in **Figure 9**; *Proteobacteria* was the most prominent phylum in these samples (69%), followed by *Firmicutes* (12%), *Actinobacteria* (7%), and *Bacteroidetes* (5%).

### NGS Analysis of Bacterial DNA in CNS Tissue From Control Subjects

**FIGURE 10:**
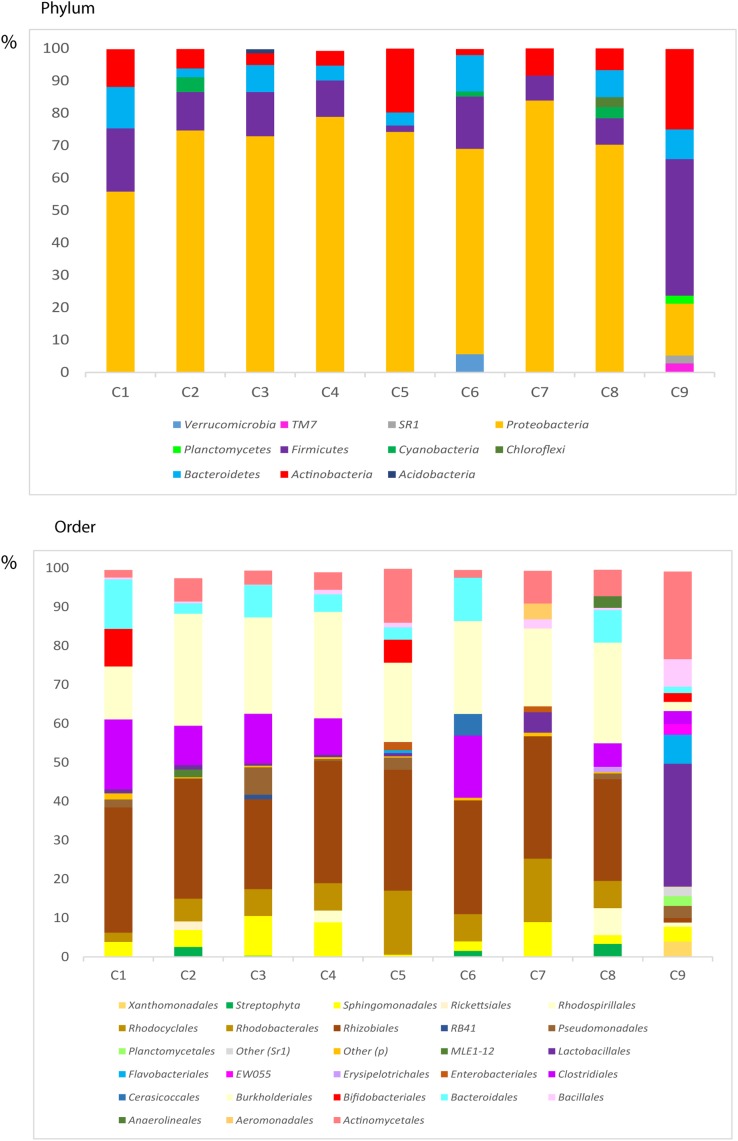
Distribution of bacteria phyla and orders obtained by NGS of DNA from control subjects. Computational analyses of the sequences obtained on the Illumina platform using Qiime classified the data into bacteria phyla and orders. **(Upper)** Shows the results of bacteria phyla obtained from ERH samples of nine control subjects. **(Lower)** Shows the results of bacterial orders obtained from ERH samples of nine controls.

One of the most surprising observations in recent years is that a variety of bacterial species can be detected in brain tissue from control subjects using NGS technology (Branton et al., 2016; Emery et al., 2017). The possibility that bacterial DNA contaminated the samples during the procedures was discarded by these groups. We extended our analyses by analyzing DNA extracted from ERH samples from 9 control subjects using NGS. The bacterial species found through this analysis are listed in Supplementary Table S8, and the bacterial phyla and orders are depicted in **Figure 10**. Close inspection of these taxa suggests that their percentages are similar to those found in AD patients. However, as indicated previously, the amount of bacterial infection could be higher in AD brains as compared with controls, although the exact bacterial taxa may not differ between AD and control brains. Nevertheless, it is possible that the presence of fungal infection in AD brains facilitates the growth of bacterial cells.

**FIGURE 11:**
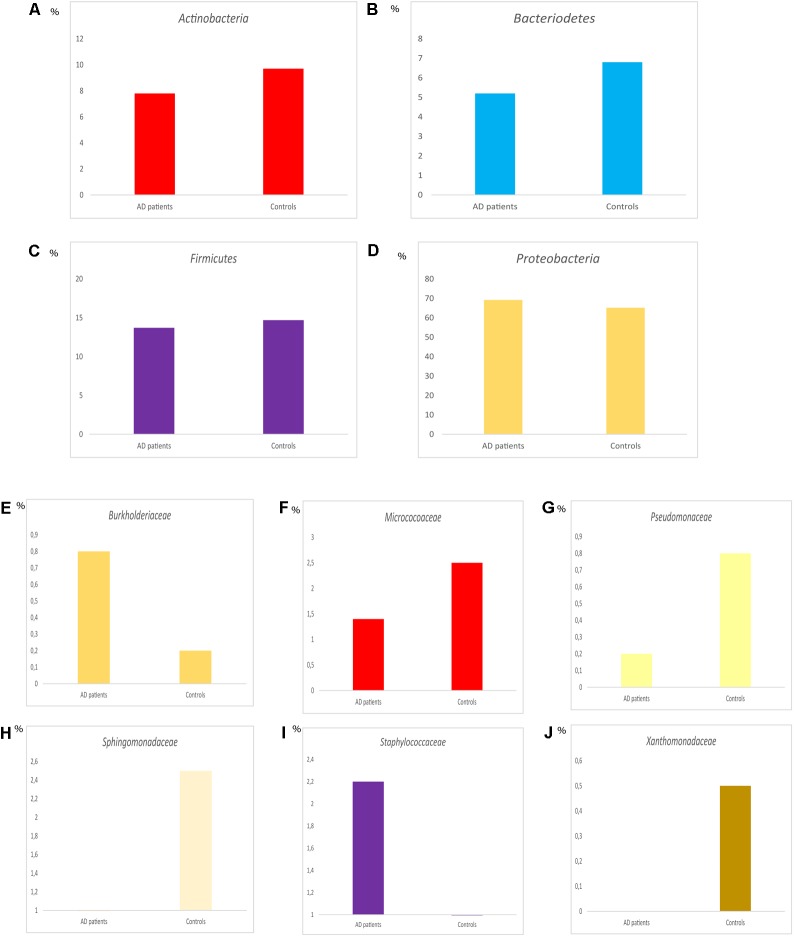
Distribution of bacteria phyla and families between AD patients and controls. Representation of median percentage of the median values of the percentages of different phyla **(A–D)**. Representation of the median of the percentages of different families **(E–J)**.

**FIGURE 12:**
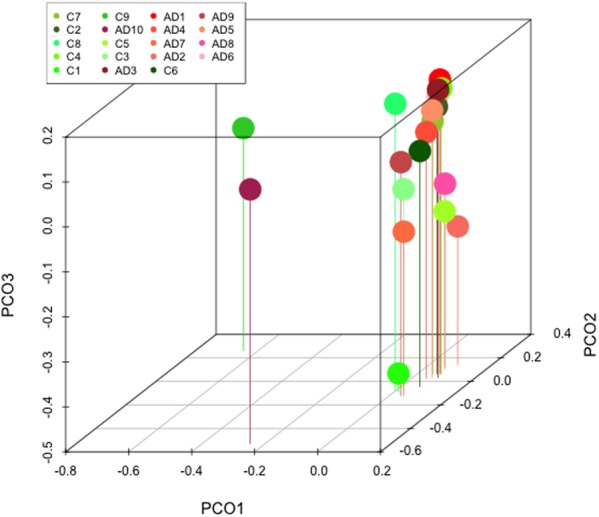
Principal component analysis of AD and control samples: 3D principal component analysis scatter plots of bacteria from ten AD patients and nine controls. The UniFrac method was used to calculate this parameter.

Comparison of the percentages of the four most relevant phyla in AD and control subjects is shown in **Figures 11A–D**. No major differences were found between the two groups. However, comparison of the percentages of some representative families revealed several variations between AD and controls (**Figures 11E–J**). For instance, *Burkholderiaceae, and Staphylococcaceae* were more prominent in AD than in controls, whereas the percentages of *Micrococcaceae, Pseudomonadaceae, Sphingomonadaceae*, and *Xanthomonadaceae* were higher in controls than in AD patients. It might be possible that the increase in the percentage of more pathogenic bacteria in the CNS in AD contributes to worsen the clinical symptoms of the disease. Finally, PCA reflected that the two groups locate in a similar position (**Figure 12**), indicating that in this regard no major differences are observed in the bacterial microbiota detected in the CNS of both groups.

We believe that these data add further support to the concept that bacterial cells exist in brain tissue. In addition, these data may serve as a springboard to uncover the microbiota of the human CNS and delineate the polymicrobial infections that appear in AD brains.

## Discussion

### Fungi and Bacteria Co-infections in AD Brains

There is mounting evidence supporting the idea that AD can be caused by microbial infections (Itzhaki, 2014; Harris and Harris, 2015; Miklossy, 2016). However, most of these studies were orientated to search for viruses or bacteria, and fungi were not considered. Nevertheless, it has been possible to detect microbial genomes in AD brain tissue with the development of very sensitive techniques such as nested PCR or NGS. To our knowledge, we are the only group that has advanced the idea that AD brains are infected by a variety of fungi (Carrasco et al., 2017). Our extensive evidence demonstrates that brain tissue from AD patients is infected by several fungal species (Alonso et al., 2014a, 2017a). Indeed, mycotic yeast and hyphal structures can be directly observed in the neural tissue, with some located intracellularly, indicating that the infection took place when the cells were still alive (Pisa et al., 2015b, 2017). Further evidence that mycoses occurred long before the death of AD patients comes from the analysis of the protein composition of *corpora amylacea*, glycoproteinaceous structures that appear in the brain with aging and are more abundant in neurodegenerative disorders. Our study revealed that fungal proteins are recruited during the formation of these bodies in the CNS of living AD patients (Pisa et al., 2016). Since these bodies need months or even years for their formation, the fungal proteins that form part of them were recruited long before death. This observation demonstrates that these mycoses existed when the patient was alive and are not the result of post-mortem contamination.

We previously reported the mycobiome in ERH tissue from AD brains by NGS (Alonso et al., 2017a), which represents the first attempt in this new field of research. Our present work extends and builds on these results by analyzing samples from the frontal region of the brain cortex (FC) of AD, revealing in more detail the mycobiome that exists in the CNS of AD patients. Importantly, our present study also reports on the mycobiome of control subjects. Accordingly, we provide the first evidence that fungal DNA belonging to a number of species can be evidenced in CNS samples from apparently normal, control subjects. Nevertheless, the burden of these mycoses is not only higher in AD brains, but also the percentages of representative genera differ from control subjects. Our results support the emerging picture that infection can increase throughout the lifetime of an individual, but it may not give rise to clinical symptoms if it remains below a given threshold. It is possible that dysbiosis of the mycobiota or the total microbiota due to diet, lifestyle, diminished immune system, among others, could trigger the increase of fungal growth in the CNS, leading to progressive AD symptoms (Daulatzai, 2014; Jiang et al., 2017). Future work in this new field of research considering the fungal hypothesis should shed more light on the origin and progression of AD.

In addition to the principal mycotic infection, bacteria also appear in the CNS of these patients, pointing to the concept that polymicrobial infections occur in AD patients (Pisa et al., 2017). It is possible that the dissemination of the primary mycosis to different regions of the CNS facilitates the growth of opportunistic microbes. Clinical trials will be necessary to understand the contribution of the fungal and bacterial infections in the development of AD. In this regard, it is of note that some antibiotics such as minocycline, as well as doxycycline and rifampin, have beneficial effects during the early stages of AD (Loeb et al., 2004; Kim and Suh, 2009). Moreover, some antibacterials, including tetracyclines and rifampin, exhibit partial antifungal activity (Lew et al., 1977; Vazquez, 1979; He et al., 2017). To our knowledge, no clinical trials with approved antifungal compounds or, better still, antifungals in combination with antibacterial agents, have been performed with AD patients.

### Microbiota Influences the Development of AD

Some animal studies have suggested a role for the gut microbiota in AD-related pathogenesis (Harach et al., 2017; Jiang et al., 2017), and the human microbiota has also been implicated as a risk factor or as an important etiological agent in neurodegenerative diseases (Tremlett et al., 2017). Indeed, different parts of the human body contain different microbiomes, which consist of a great diversity of bacteria and fungi (Huffnagle and Noverr, 2013) that can vary between individuals and may change throughout the lifetime. Only 0.1–1.0% of the microbiome, however, corresponds to fungi (Qin et al., 2010). Yet, the mycobiome includes over 390 species that are present in different anatomical locations, such as skin and mucoses, the respiratory tract, the oral cavity and the digestive tract (Cui et al., 2013; Dupuy et al., 2014; Gouba and Drancourt, 2015). Some studies have reported that 335 fungal species belonging to 158 genera can be found in the human digestive tract and the oral cavity (Ghannoum et al., 2010; Gouba and Drancourt, 2015). Notably, most of these species cannot be cultured and must be identified by molecular techniques, such as PCR or NGS. Thus, from the 247 species found in the digestive tract, only 59 were could grow in culture and the remaining species were identified by molecular analysis (Findley et al., 2013; Gouba and Drancourt, 2015). It should be possible that at least some of these species could enter the human body and colonize internal tissues.

### Portal of Entry of Microbes to the CNS

The identification of fungi and bacteria in the CNS of AD patients raises the question as to how these polymicrobial infections colonize the neural tissue. One possibility is that this colonization may arise from the gut microbiota that passes the gastrointestinal mucosa and reaches the blood stream. Subsequently, these microbes would be disseminated to other organs and tissues of the human body, including the nervous system. Indeed, a number of bacteria have been identified in blood from healthy persons (Paisse et al., 2016). Another portal of entry could be the oral cavity and the nasopharyngeal region; the microbiota could reach the olfactory nerve, spreading to the olfactory bulb and the entorhinal area (Olsen and Singhrao, 2015). Of note, the oral microbiota, as well as inadequate oral hygiene, have been suggested as important contributors to AD etiology (Gaur and Agnihotri, 2015). The repeated passage of diverse microorganisms into the bloodstream could initiate the colonization of some blood vessels. The colonization of discrete areas of a given tissue by fungi could evade the immune system by a variety of strategies (Kong and Jabra-Rizk, 2015; Marcos et al., 2016). For example, the continuous production of massive amounts of fungal polysaccharides in a discrete region of a tissue could block an attack by immune cells. The infection could then spread to neighboring areas and, importantly, could facilitate the secondary colonization by other fungi or bacteria. This local depression of the immune system would lead to the formation of discrete infectious foci, followed by the slow distribution to other tissues. Therefore, in our model of polymicrobial infection in AD patients, not only is the CNS subject to microbial attack, but signs of disseminated microbial colonization could exist in other organs. Consistent with this model is the finding that increased levels of inflammatory cytokines is detected in patient serum years before the diagnosis of the disease (Brosseron et al., 2014; King and Thomas, 2017; Lai et al., 2017). Analysis of other tissues by NGS may unveil a “tissular microbiome” of internal organs of the human body. A comprehensive study of the fungi and bacteria that could colonize these organs will provide insights into the different susceptibilities for microbiomes depending on the genetic background of the individual.

### Polymicrobial Infections and Human Diseases

The modification of the microbiota from the intestinal tract or from other parts of the human body can influence the development of a variety of diseases, such as multiple sclerosis, intestinal bowel disease, rheumatoid arthritis and autoimmune diseases (Scher et al., 2013; Jangi et al., 2016; Hall et al., 2017; Ni et al., 2017). Interestingly, a variety of bacteria have been identified in human tissue of several chronic systemic pathologies, including arthritis, atherosclerosis, biliary cirrhosis and aortic aneurysms (Padgett et al., 2005; Marques da Silva et al., 2006; Ott et al., 2006; Koren et al., 2011; Leech and Bartold, 2015). It is possible that many of the diseases known as “autoimmune diseases” are in fact microbial infections that remain hidden to routine analytical tests. The low level colonization of a given tissue by several microbes can be revealed by the techniques described in this work: immunohistochemistry using specific antibodies and nested PCR or NGS. In these cases, the immune system detects the infection, which is interpreted as an attack on the host tissue. Therefore, the host immune system recognizes these microbes and tries to eradicate it by stimulating the production of cytokines. This immune attack is counteracted by different microorganisms, leading to the establishment of a chronic and progressive polymicrobial infection. Although some investigations have attempted to identify bacteria in internal human tissues (other than the gut), the analysis of fungi remains understudied. Future work directed to analyze microbial colonization in human tissues, including the CNS, may change the present concepts about a number of human pathologies.

## Author Contributions

DP and AF-F performed the immunohistochemistry analyses. RA carried out the PCR and NGS analysis. LC designed the study and wrote the paper. All authors discussed the results and commented on the manuscript.

## Conflict of Interest Statement

The authors declare that the research was conducted in the absence of any commercial or financial relationships that could be construed as a potential conflict of interest.

## Footnotes

1 http://www.ebi.ac.uk/ena/data/view/PRJEB23885

2 http://www.bioinformatics.babraham.ac.uk/projects/fastqc/

3 http://nbviewer.ipython.org/github/biocore/qiime/blob/1.9.1/examples/ipynb/Fungal-ITS-analysis.ipynb

4 ftp://ftp.microbio.me/qiime/tutorial_files/its_12_11_otus.tgz

5 http://qiime.org/
