# Supplementary material for: Infection of Fungi and Bacteria in Brain Tissue From Elderly Persons and Patients With Alzheimer’s Disease
Source: Front Aging Neurosci. 2018 May 24;10:159. doi: 10.3389/fnagi.2018.00159 (PMC5976758; doi:10.3389/fnagi.2018.00159)
Supplement: Supplementary file 1 [file Table_1.pdf]

Supplementary table I. Summary of AD patients and controls subjects.

Patients	Age	Gender	Brain region	Braak TAU
AD1	80	Female	FC and ERH	5
AD2	84	Female	FC and ERH	5
AD3	79	Female	FC and ERH	6
AD4	81	Female	FC and ERH	6
AD5	87	Male	FC and ERH	5
AD6	92	Male	FC and ERH	6
AD7	81	Male	FC and ERH	5
AD8	87	Female	FC and ERH	6
AD9	86	Female	FC and ERH	5
AD10	62	Male	FC and ERH	5
AD11	83	Female	ERH	Unknown
Controls				Systemic diseases
C1	56	Male	ERH	Unknown
C2	48	Female	ERH	Unknown
C3	63	Male	ERH	Adenocarcinoma of the pancreas
C4	55	Female	ERH	Arterial hypertension
C5	62	Female	ERH	Myolodysplastic syndrome
C6	84	Male	ERH	Cardiomyopathy
C7	37	Female	ERH	Sepsis
C8	54	Male	ERH	Ischemic cardiopathy
C9	59	Female	ERH	Acute pancreatitis
C10	53	Male	FC<br>ERH<br>MD<br>SC	Unknown
C11	78	Male	FC<br>ERH<br>MD<br>SC	Unknown
C12	74	Female	FC<br>ERH<br>MD<br>SC	Unknown
C13	83	Female	FC<br>ERH<br>MD<br>SC	Unknown
C14	89	Male	GFS	Urosepsis
C15	90	Female	GFS	Possible infection
C16	87	Male	CP	Unknown

FC: Frontal cortex; ERH : Entorhinal cortex; MD: Medulla; SC: Spinal cord; GFS: Superior Frontal cortex; PC: Parietal cortex
