# Supplementary material for: Infection of Fungi and Bacteria in Brain Tissue From Elderly Persons and Patients With Alzheimer’s Disease
Source: Front Aging Neurosci. 2018 May 24;10:159. doi: 10.3389/fnagi.2018.00159 (PMC5976758; doi:10.3389/fnagi.2018.00159)
Supplement: Supplementary file 2 [file Table_2.pdf]

Supplementary table II. Representation of fungal species over 1 % from Frontal cortex brain region of ten AD patient

**AD1**		**AD2**		**AD3**		**AD4**		**AD5**	
Original paired reads :213462		Original paired reads :260368		Original paired reads : 248062		Original paired reads :223155		Original paired reads : 209034	
Joined sequences (%): 97.9		Joined sequences (%): 98.4		Joined sequences (%): 97.8		Joined sequences (%): 97.8		Joined sequences (%): 98.6	
Chromelosporium carneum	65.9	*Uncultured fungus clone S24T_41*	18.4	*Uncultured fungus clone S24T_41*	21.5	*Uncultured fungus clone S24T_41*	16.6	*Uncultured fungus clone S24T_41*	20.9
Malassezia_sp_HM_2008	12.8	*Xylaria curta*	9.7	*Emericella nidulans*	16.8	*Pleurothecium_sp_LXS_2012*	16.5	*Uncultured Basidiomycota*	11.0
Uncultured fungus clone S24T_41	5.4	*Uncultured Basidiomycota*	8.2	*Candida deformans*	8.7	*Candida deformans*	6.7	*Candida deformans*	8.5
Candida deformans	2.2	*Candida deformans*	7.5	*Botrytis cinerea*	1.6	*Uncultured Basidiomycota*	1.7	*Davidiella tassiana*	6.4
		Uncultured soil fungus	5.5	*Uncultured fungus clone S110T_52*	1.3	*Botrytis cinerea*	1.2	*Cryptococcus magnus*	2.9
		Candida zeylanoides	3.1	*Uncultured Basidiomycota*	1.1	*Uncultured fungus clone S110T_52*	1.0	*Botrytis cinerea*	1.6
		Botrytis cinerea	1.4	*Alternaria alternata*	1.5	*Alternaria alternata*	1.1	*Alternaria alternata*	1.4
		Uncultured fungus clone S110T_52	1.1					*Uncultured fungus clone S110T_52*	1.3

**AD6**		**AD7**		**AD8**		**AD9**		**AD10**	
Original paired reads : 241821		Original paired reads : 210396		Original paired reads :359221		Original paired reads :147296		Original paired reads : 361090	
Joined sequences (%): 98.1		Joined sequences (%): 98.2		Joined sequences (%): 97.2		Joined sequences (%): 97.7		Joined sequences (%): 97.8	
Uncultured fungus clone S24T_41	25.5	*Uncultured Basidiomycota*	19.3	*Uncultured fungus clone S24T_41*	20.8	*Uncultured fungus clone S24T_41*	22.2	*Emericella nidulans*	35.4
Candida deformans	10.4	*Uncultured fungus clone S24T_41*	15.7	*Candida deformans*	8.5	*Emericella nidulans*	9.1	*uncultured Basidiomycota*	32.8
Uncultured Basidiomycota	4.2	*Candida deformans*	6.4	*Basidiomycota_sp_D3*	3.9	*Candida deformans*	9.1	*Xeromyces bisporus*	23.3
Botrytis cinerea	1.9	*Botrytis cinerea*	1.2	*Botrytis cinerea*	1.6	*Candida parapsilosis*	3.2		
Alternaria alternata	1.7	*Alternaria alternata*	1.1	Uncultured fungus clone S110T_52	1.3	*Acremonium_sp_JJP_2009a*	2.7		
Uncultured fungus clone S110T_52	1.6			*Uncultured Basidiomycota*	1.0	*Botrytis cinerea*	1.7		
Uncultured Basidiomycota	1.3			*Alternaria alternata*	1.4	Uncultured fungus clone S110T_52	1.4		
uncultured malassezia	1.1					*Uncultured Basidiomycota*	1.1		
Alternaria tenuissima	1.1					*Alternaria alternata*	1.5		

Supplementary table II. Representation of fungal species over 1 % from Frontal cortex brain region of ten AD patient
