# Supplementary material for: Infection of Fungi and Bacteria in Brain Tissue From Elderly Persons and Patients With Alzheimer’s Disease
Source: Front Aging Neurosci. 2018 May 24;10:159. doi: 10.3389/fnagi.2018.00159 (PMC5976758; doi:10.3389/fnagi.2018.00159)
Supplement: Supplementary file 3 [file Table_3.pdf]

Supplementary table III. Common fungal species detected by Sanger and NGS from ten AD patients.

Patients	Common species
AD1	*Cryptococcus magnus, Cladosporium sp, Uncultured malassezia*
AD2	*Cladosporium sp*
AD3	*Uncultured malassezia*
AD4	
AD5	*Alternaria alternata*
AD6	
AD7	*Cladosporium sp*
AD8	*Malassezia globosa, Uncultured malassezia*
AD9	*Uncultured aureibasidium*
AD10	*Sporobolomyces sp*
