# Supplementary material for: Infection of Fungi and Bacteria in Brain Tissue From Elderly Persons and Patients With Alzheimer’s Disease
Source: Front Aging Neurosci. 2018 May 24;10:159. doi: 10.3389/fnagi.2018.00159 (PMC5976758; doi:10.3389/fnagi.2018.00159)
Supplement: Supplementary file 4 [file Table_4.pdf]

Supplementary table IV. Common genera obtained by NGS in samples of ERH and FC from AD patients.

Patients number in this work FC	Patients number in previous work ERH	Common genera
AD1	ND	
AD2	AD2	*Alternaria,Botrytis, Candida, Malassezia*
AD3	AD3	*Alternaria,Botrytis, Candida, Malassezia*
AD4	AD4	*Alternaria,Botrytis, Candida, Malassezia*
AD5	AD5	*Alternaria, Basidiomycota, Botrytis,Davidiella,Malassezia*
AD6	AD6	*Alternaria,Botrytis, Candida, Malassezia*
AD7	AD7	*Alternaria, Botrytis,Candida,Malassezia*
AD8	AD8	*Alternaria, Botrytis,Candida,Malassezia*
AD9	ND	
AD10	AD9	

ND: Not done
