# Supplementary material for: Infection of Fungi and Bacteria in Brain Tissue From Elderly Persons and Patients With Alzheimer’s Disease
Source: Front Aging Neurosci. 2018 May 24;10:159. doi: 10.3389/fnagi.2018.00159 (PMC5976758; doi:10.3389/fnagi.2018.00159)
Supplement: Supplementary file 5 [file Table_5.pdf]

Supplementary table V. Representation of fungal species over 1 % from twelve control brains.

C1	C2	C3	C4	C5	C6
Original paired reads:233188<br>Joined sequences(%):92.8	Original paired reads:219891<br>Joined sequences(%):97.8	Original paired reads:247621<br>Joined sequences(%):98.8	Original paired reads:187800<br>Joined sequences(%):97.7	Original paired reads:205726<br>Joined sequences(%):98.1	Original paired reads:204983<br>Joined sequences(%):95.4
Candida deformans 21.9	Candida deformans 16.1	Fusarium oxysporum 35.2	Fusarium oxysporum 26.6	Aspergillus niger 20.5	Aspergillus penicillioides 33.9
Uncultured Phoma 13.1	Uncultured Ascomycota 12.1	Aspergillus niger 12.8	Candida deformans 12.6	Candida deformans 11.5	Candida deformans 12.6
Botrytis cinerea 12.9	Botrytis cinerea 9.6	Candida deformans 11.5	Acremonium_sp_JJP_2009a 8.2	Uncultured Basidiomycota 11.1	Botrytis cinerea 7.5
Uncultured fungus clone 038A45644 6.7	Uncultured Phoma 9.6	Uncultured Phoma 6.9	Botrytis cinerea 7.5	Acremonium_sp_JJP_2009a 10.4	Uncultured Phoma 7.5
Uncultured fungus clone S24T_41 5.6	Rhodotorula minuta 8.9	Botrytis cinerea 6.8	Uncultured Phoma 7.5	Uncultured Phoma 6.9	Uncultured Basidiomycota 5.1
Uncultured fungus clone MOTU_4160 2.3	Rhizophydium_sp_PL_157 6.6	Uncultured fungus clone 038A45644 3.5	Uncultured fungus clone 038A45644 6.8	Botrytis cinerea 6.8	Cladonia fimbriata 4.1
	Uncultured fungus clone 038A45644 4.9	Uncultured fungus clone S24T_41 2.9	Uncultured fungus clone S24T_41 3.3	Uncultured fungus clone 038A45644 3.5	Uncultured fungus clone 038A45644 3.8
	Uncultured fungus clone S24T_41 4.2	Uncultured Basidiomycota 1.7	Uncultured Chytridiomycota 3.0	Uncultured fungus clone S24T_41 3.0	Uncultured fungus clone S24T_41 3.3
	Uncultured fungus clone MOTU_4160 1.7	Uncultured fungus clone MOTU_4160 1.2	Uncultured fungus clone MOTU_4160 1.3	Uncultured fungus clone MOTU_4160 1.2	Uncultured fungus clone MOTU_4160 1.3

C7	C8	C9	C14	C15	C16
Original paired reads:833385<br>Joined sequences(%):98.7	Original paired reads:224413<br>Joined sequences(%):97.2	Original paired reads:184818<br>Joined sequences(%):96.3	Original paired reads:200703<br>Joined sequences(%):97.3	Original paired reads:300056<br>Joined sequences(%):97.1	Original paired reads:336317<br>Joined sequences(%):97.7
Candida deformans 17.1	Davidiella tassiana 18.6	Candida deformans 18.7	Candida deformans 45.1	Candida deformans 36.1	Candida deformans 44.7
uncultured Ascomycota 8.3	Candida deformans 10.5	Botrytis cinerea 11.1	unculturedsoil_fungus 12.1	Botrytis cinerea 4.7	Candida_sp 11.2
Botrytis cinerea 4.6	Uncultured Phoma 6.3	Uncultured Phoma 11.1	Botrytis cinerea 5.9	Uncultured fungus clone S24T_41 3.8	Davidiella_tassiana 7.1
Uncultured Phoma 4.6	Botrytis cinerea 6.2	Uncultured fungus clone 038A45644 5.7	Uncultured fungus clone S24T_41 4.7	Uncultured fungus clone S346 2.3	Botrytis cinerea 5.9
Uncultured fungus clone S24T_41 4.4	Malassezia_sp_HM_2008 3.8	Uncultured fungus clone S24T_41 4.8	Uncultured fungus clone S346 2.9		Uncultured fungus clone S24T_41 4.7
unculturedBasidiomycota 4.3	Fungal_endophyte_sp_O26_3333 3.8	Uncultured fungus clone MOTU_4160 2.0			Uncultured fungus clone S346 2.8
Candida_sp, 4.0	Uncultured fungus clone 038A45644 3.2	Acremonium_sp_JJP_2009a 1.9			
Cryptococcus magnus 2.7	Uncultured fungus clone S24T_41 2.7				
Davidiella tassiana 2.3	Fusarium oxysporum 2.6				
Uncultured fungus clone 038A45644 2.3	Ascomycota_sp_I406 2.5				
	Uncultured fungus clone MOTU_4160 1.2				
