# Supplementary material for: Infection of Fungi and Bacteria in Brain Tissue From Elderly Persons and Patients With Alzheimer’s Disease
Source: Front Aging Neurosci. 2018 May 24;10:159. doi: 10.3389/fnagi.2018.00159 (PMC5976758; doi:10.3389/fnagi.2018.00159)
Supplement: Supplementary file 6 [file Table_6.pdf]

Supplementary table VI. . Representation of fungal species > 1% from four regions of four control brains.

C10 FC		C10 ERH		C10MD		C10SC	
Originalpairedreads:178721<br>Joined sequences(%):98,5		Original paired reads:160135<br>Joined sequences(%):98,7		Original paired reads:207232<br>Joined sequences(%):98,3		Original paired reads:209528<br>Joined sequences(%):98,6	
Candida deformans	*23,1*	*Uncultured Ascomycota*	*36,36*	*Candida deformans*	*13,40*	*Uncultured Basidiomycota*	*22,80*
Uncultured Phoma	*13,80*	*Rhizophydiales_sp*	*12,36*	*Fusarium oxysporum*	*10,09*	*Acremonium_sp*	*12,83*
Botrytis cinerea	*12,10*	*Botrytis cinerea*	*6,6*	*Uncultured Phoma*	*8,00*	*Candida deformans*	*10,00*
Uncultured fungus clone 038A45644	*7*	*Candida deformans*	*11*	*Botrytis cinerea*	*7,00*	*Davidiella tassiana*	*8,36*
*Uncultured fungus clone S24T_*	*5,90*	*Uncultured Phoma*	*6,60*	*Uncultured fungus clone 038A45644*	*4,1*	*Phaeosphaeriaceae_sp*	*8,25*
Uncultured fungus clone MOTU_4160	*2,50*	*Uncultured fungus clone 038A45644*	*3,4*	*Uncultured fungus clone S24T_*	*3,50*	*Uncultured Phoma*	*6,00*
Uncultured Basidiomycota	*2,32*	*Uncultured fungus clone S24T_*	*2,90*	*Uncultured Basidiomycota*	*2,77*	*Botrytis cinerea*	*5,30*
		Uncultured fungus clone MOTU_4160	*1,20*	*Candida_cellae*	*1,69*	*Uncultured fungus clone 038A45644*	*3,1*
				Uncultured fungus clone MOTU_4160	*1,40*	*Uncultured fungus clone S24T_*	*2,60*
						Uncultured fungus clone MOTU_4160	*1,10*
C11FC		C11ERH		C11MD		C11SC	
Original paired reads:179243<br>Joined sequences(%):99		Original paired reads:215288<br>Joined sequences(%):97,8		Original paired reads:188692<br>Joined sequences(%):97,3		Original paired reads:200505<br>Joined sequences(%):96,6	
Uncultured Ascomycota	*76,78*	*Phoma_sp*	*16,00*	*Davidiella tassiana*	*20,36*	*Candida deformans*	*10,40*
Candida deformans	*3,20*	*Xylaria_curta*	*13,75*	*Aspergillus niger*	*11,97*	*Aspergillus niger*	*8,97*
Uncultured Phoma	*1,90*	*Penicillium aurantiogriseum*	*11,29*	*Candida deformans*	*10,5*	*Uncultured Phoma*	*6,20*
Aspergillus_niger	*1,86*	*Candida deformans*	*8,90*	*Uncultured Ascomycota*	*8,09*	*Botrytis cinerea*	*5,60*
Botrytis cinerea	*1,70*	*Davidiella_tassiana*	*7,31*	*Uncultured Phoma*	*6,30*	*Uncultured Basidiomycota*	*3,30*
Davidiella_tassiana	*1,32*	*Uncultured Phoma*	*5,40*	*Botrytis cinerea*	*5,5*	*Uncultured fungus clone 038A45644*	*3,1*
Lasiodiplodia_theobromae	*1,31*	*Botrytis cinerea*	*4,70*	*Acremonium_sp*	*4,56*	*Uncultured fungus clone S24T_*	*2,70*
Rhodotorula ingeniosa	*1,20*	*Uncultured fungus clone 038A45644*	*2,7*	*uncultured Chytridiomycota*	*4,26*	*Rhizophydiales_sp*	*2,43*
Eupenicillium_sp	*1,01*	*Uncultured fungus clone S24T_*	*2,30*	*Uncultured fungus clone 038A45644*	*3,1*	*Uncultured fungus clone MOTU_4160*	*1,10*
Uncultured fungus clone 038A45644	*1*			*Uncultured fungus clone S24T_*	*2,70*	*Candida glabrata*	*1,09*
				Uncultured fungus clone MOTU_4160	*1,10*		
C12FC		C12ERH		C12MD		C12SC	
Original paired reads:193250		Original paired reads:178600		Original paired reads:198565		Original paired reads:173107	

Joined sequences(%):98,8		Joined sequences(%):98,9		Joined sequences(%):98,9		Joined sequences(%):98,4	
Davidiella tassiana	*36,52*	*Davidiella tassiana*	*34,62*	*Davidiella tassiana*	*43,15*	*Uncultured Basidiomycota*	*32,67*
Candida deformans	*11,90*	*Acremonium_sp*	*11,32*	*Candida deformans*	*10,70*	*Davidiella tassiana*	*26,92*
Uncultured Phoma	*7,10*	*Candida deformans*	*9,80*	*Aspergillus niger*	*6,52*	*unculturedsoil fungus*	*12,13*
Botrytis cinerea	*6,2*	*Uncultured Phoma*	*5,80*	*Uncultured Phoma*	*6,40*	*Candida deformans*	*5,70*
Uncultured fungus clone 038A45644	*3,6*	*Botrytis cinerea*	*5,10*	*Botrytis cinerea*	*4,50*	*Uncultured Phoma*	*3,40*
Uncultured Rhizopogon	*3,30*	*Uncultured Basidiomycota*	*3,77*	*Uncultured fungus clone 038A45644*	*3,3*	*Botrytis cinerea*	*3,00*
*Uncultured fungus clone S24T_*	*3,10*	*root_associated_fungal_sp_EP1_6*	*3,38*	*Uncultured Rhizopogon*	*2,90*	*Acremonium sp*	*1,82*
Uncultured fungus clone MOTU_4160	*1,30*	*Rhizophydium Sp*	*3,01*	*Uncultured fungus clone S24T_*	*2,80*	*Uncultured fungus clone 038A45644*	*1,7*
Xylaria curta	*1,20*	*Uncultured fungus clone 038A45644*	*2,9*	*Acremonium_sp*	*2,34*	*Uncultured Rhizopogon*	*1,50*
		Uncultured Rhizopogon	*2,60*	*Uncultured fungus clone MOTU_4160*	*1,10*	*Uncultured fungus clone S24T_*	*1,50*
		*Uncultured fungus clone S24T_*	*2,50*				
		unculturedsoil_fungus	*1,28*				
		Uncultured fungus clone MOTU_4160	*1,00*				
C13FC		C13ERH		C13MD		C13SC	
Original paired reads:150780		Original paired reads:175759		Original paired reads:190566		Original paired reads:178420	
Joined sequences(%):98,6		Joined sequences(%):98,9		Joined sequences(%):98		Joined sequences(%):97,5	
Xylaria curta	*21,83*	*Davidiella tassiana*	*44,76*	*Aspergillus niger*	*11,69*	*Davidiella tassiana*	*38,87*
Davidiella tassiana	*13,54*	*Candida deformans*	*11,10*	*Candida deformans*	*11,50*	*Candida deformans*	*14,20*
Anthracoidea_aspera	*12,28*	*Uncultured Phoma*	*6,60*	*uncultured Basidiomycota*	*9,71*	*Uncultured Phoma*	*8,50*
unculturedsoil_fungus	*8,83*	*Botrytis cinerea*	*5,80*	*Uncultured Phoma*	*6,90*	*Botrytis cinerea*	*7,5*
Candida deformans	*6,10*	*Uncultured fungus clone 038A45644*	*3,4*	*Acremonium_sp*	*6,85*	*Uncultured fungus clone 038A45644*	*4,3*
Uncultured Phoma	*3,60*	*Uncultured Rhizopogon*	*3,00*	*Botrytis cinerea*	*6,00*	*Uncultured Rhizopogon*	*3,80*
Botrytis cinerea	*3,10*	*Uncultured fungus clone 41*	*2,90*	*Uncultured fungus clone 038A45644*	*3,5*	*Uncultured fungus clone S24T_*	*3,70*
Physoderma_maydis	*2,94*	*Xylaria curta*	*2,13*	*Uncultured Rhizopogon*	*3,00*	*Uncultured fungus clone MOTU_4160*	*1,50*
Uncultured fungus clone 038A45644	*1,80*	*Phoma_sp*	*1,93*	*Uncultured fungus clone S24T_*	*3,00*		
Aspergillus caesiellus	*1,77*			*Uncultured fungus clone MOTU_4160*	*1,20*		
Uncultured Rhizopogon	*1,60*			*Uncultured soil fungus*	*8,3*		
*Uncultured fungus clone S24T_*	*1,60*						
