# Supplementary material for: Infection of Fungi and Bacteria in Brain Tissue From Elderly Persons and Patients With Alzheimer’s Disease
Source: Front Aging Neurosci. 2018 May 24;10:159. doi: 10.3389/fnagi.2018.00159 (PMC5976758; doi:10.3389/fnagi.2018.00159)
Supplement: Supplementary file 7 [file Table_7.pdf]

Supplementary table VII. Bacterial species detected by NGS in ten AD patients.

**AD1**		**AD2**		**AD3**		**AD4**		**AD5**	
Number of reads :224151		Number of reads:210019		Number of reads:185208		Number of reads:204746		Number of reads:206408	
Sequences joined : 95%		Sequences joined :94,8%		Sequences joined :95,3%		Sequences joined:95%		Sequences joined:92,9%	
Methylobacteriaceae	21.0	*Methylobacteriaceae*	33.6	*Cupriavidus*	17.4	*Methylobacteriaceae*	22.7	*Methylobacteriaceae*	17.9
Cupriavidus	13.3	*Bradyrhizobiaceae*	11.1	*Bradyrhizobiaceae*	12.5	*Bradyrhizobiaceae*	9.6	*Cupriavidus*	11.2
Staphylococcus	8.1	*Cupriavidus*	10.1	*Streptophyta*	12.0	*Cupriavidus*	8.1	*Sphingomonas*	9.2
Zoogloea	7.8	*Bifidobacterium adolescentis*	6.7	*Methylobacteriaceae*	10.7	*Zoogloea*	5.2	*Sphingomonadales*	6.8
Azospira	6.9	*Sphingomonas*	4.7	*Comamonadaceae*	6.6	*Acidovorax defluvii*	4.9	*Prevotella copri*	4.7
Comamonadaceae	5.8	*Escherichia coli*	3.1	*Sphingomonas*	5.1	*Eubacterium biforme*	4.8	*Comamonadaceae*	4.7
Ruminococcus	4.3	*Acidovorax defluvii*	3.1	*Roseburia*	4.3	*Ruminococcaceae*	4.5	*Acidimicrobiales*	4.4
Sphingomonadales	3.8	*Zoogloea*	3.0	*Sphingomonadales*	3.6	*Sphingomonadales*	3.9	*Bradyrhizobiaceae*	4.4
Corynebacterium	2.5	*Azospira*	2.5	*Rothia dentocariosa*	3.4	*Sphingomonas*	3.7	*Parabacteroides*	4.3
Acidovorax defluvii	2.5	*Halomonas*	2.2	*Staphylococcus*	3.1	*Roseburia*	3.5	*Acidovorax defluvii*	4.2
Kineosporiaceae	2.0	*Lachnospiraceae*	2.1	*Acidovorax defluvii*	2.3	*Veillonella dispar*	2.9	*Azospira*	3.3
Bradyrhizobiaceae	1.9	*Micrococcus luteus*	1.8	*Roseburia faecis*	2.1	*Rhodobacter*	2.2	*Prevotella*	2.5
Micrococcus luteus	1.8	*Corynebacterium*	1.3	*Azospira*	1.9	*ZB2*	2.1	*Cystobacterineae*	2.3
Burkholderiales	1.4	*Ralstonia*	1.3	*Lachnospiraceae*	1.5	*Dialister*	2.1	*Staphylococcus*	2.0
Comamonadaceae	1.2			*Rikenellaceae*	1.4	*Streptococcus*	1.7	*Prevotella nigrescens*	1.8
Rothia dentocariosa	1.2			*Propionibacterium acnes*	1.0	*Prevotella copri*	1.3	*Rikenellaceae*	1.8
Acetobacteraceae	1.0			*ZB2*	1.0	*Methylobacterium*	1.2	*Lachnospira*	1.7
Rhizobiales	1.0					*Phascolarctobacterium*	1.2	*Rhodoplanes elegans*	1.1
						Hyphomonadaceae	1.1		

**AD6**		**AD7**		**AD8**		**AD9**		**AD10**	
Number of reads :223350		Number of reads :192670		Number of reads :190446		Number of reads :205687		Number of reads :256223	
Sequences joined : 94,8%		Sequences joined : 94,7%		Sequences joined : 94,8%		Sequences joined : 94,7%		Sequences joined : 94,4%	
Methylobacteriaceae	24.2	*Methylobacteriaceae*	25.1	*Methylobacteriaceae*	21.9	*Methylobacteriaceae*	17.7	*Prevotella copri*	14.7
Cupriavidus	17.8	*Azospira*	12.,9	*Cupriavidus*	11.6	*Cupriavidus*	16.2	*Lachnospira*	12.6
Corynebacterium	6.1	*Streptophyta*	8.7	*Comamonadaceae*	8.6	*Bradyrhizobiaceae*	9.7	*Comamonadaceae*	4.0
Bradyrhizobiaceae	5.9	*Cupriavidus*	7.0	*Bifidobacterium adolescentis*	6.5	*Prevotella copri*	8.3	*Coriobacteriaceae*	3.9
Burkholderia	5.0	*Mitochondria*	6.5	*Escherichia coli*	6.3	*Comamonadaceae*	5.6	*Staphylococcus*	3.9
Rhodoplanes elegans	4.4	*Faecalibacterium prausnitzii*	5.8	*Bradyrhizobiaceae*	5.5	*Catenibacterium*	5.2	*Roseburia*	3.8
Ruminococcus bromii	3.7	*Prevotella copri*	3.7	*Succinivibrio*	5.1	*Thermotogaceae*	5.0	*Faecalibacterium prausnitzii*	3.8
Gemellaceae	3.4	*Acidovorax defluvii*	3.1	*Sphingomonadales*	4.2	*Azospira*	4.2	*Achromobacter*	3.8
Sphingomonadales	3.0	*Comamonadaceae*	2.7	*Staphylococcus*	2.6	*Rubellimicrobium*	4.1	*Methylobacteriaceae*	3.7
Comamonadaceae	2.6	*Anaerococcus*	2.5	*Chitinophagaceae*	2.5	*Acidovorax defluvii*	3.9	*Escherichia coli*	3.5
Corynebacterium kroppenstedtii	2.5	*Bradyrhizobiaceae*	2.5	*Azospira*	2.3	*Streptococcus*	3.3	*Rhizobiales*	3.4
Micrococcus luteus	2.5	*Corynebacterium*	1.9	*A17*	1.9	*Amaricoccus*	2.6	*Cupriavidus*	3.4
Methylobacterium	1.8	*Sphingomonas*	1.6	*Corynebacterium kroppenstedtii*	1.8	*Anaerococcus*	2.1	*Dialister*	3.3
Azospira	1.8	*Methylobacterium*	1.3	*Sphingomonas*	1.7	*Finegoldia*	1.3	*Sutterella*	3.1
Finegoldia	1.7	*Betaproteobacteria*	1.2	*Exiguobacterium*	1.4			*Eubacterium biforme*	2.8
Acidovorax defluvii	1.4	*Streptococcus*	1.1	*TM7-3*	1.4			*Corynebacterium kroppenstedtii*	2.4
Propionibacterium acnes	1.3			*Acetobacteraceae*	1.2			*Streptococcus anginosus*	2.0
Sphingomonas	1.1			*Fusobacterium*	1.2			*Streptococcus*	1.9
				BA008	1.1			*Azospira*	1.9
				Burkholderiales	1.0			*Kineosporiaceae*	1.5
								Ruminococcaceae	1.4
								Corynebacterium kroppenstedtii	1.3
								Propionibacterium acnes	1.2
								Acidovorax defluvii	1.1

				Burkholderiales	1.1
