# Supplementary material for: Infection of Fungi and Bacteria in Brain Tissue From Elderly Persons and Patients With Alzheimer’s Disease
Source: Front Aging Neurosci. 2018 May 24;10:159. doi: 10.3389/fnagi.2018.00159 (PMC5976758; doi:10.3389/fnagi.2018.00159)
Supplement: Supplementary file 8 [file Table_8.pdf]

Supplementary table VIII. Bacterial species detected by NGS in nine controls.

C1		C2		C3		C4		C5	
Number of reads:178739<br>Sequences joined:93,5%		Number of reads:185748<br>Sequences joined:94,6%		Number of reads:170630<br>Sequences joined:94,6%		Number of reads:194306<br>Sequences joined:94,8%		Number of reads:195484<br>Sequences joined:95,5%	
Methylobacteriaceae	26.5	*Comamonadaceae*	19.2	*Methylobacteriaceae*	15.0	*Methylobacteriaceae*	22.7	*Methylobacteriaceae*	23.2
SMB53	7.4	*Methylobacteriaceae*	18.0	*Cupriavidus*	11.9	*Cupriavidus*	15.7	*Arthrobacter oxydans*	10.4
Prevotella stercorea	7.4	*Bradyrhizobiaceae*	8.1	*Acidovorax defluvii*	8.5	*Comamonadaceae*	7.9	*Cupriavidus*	7.3
Bifidobacterium adolescentis	7.2	*Cupriavidus*	6.4	*Sphingomonas*	7.7	*Sphingomonadales*	6.8	*Comamonadaceae*	6.4
Cupriavidus	5.8	*Dialister*	4.8	*Azospira*	6.8	*Azospira*	5.3	*Paracoccus*	6.0
Prevotella copri	4.5	*Sphingomonas*	3.5	*Roseburia*	4.8	*Bacteroidales*	4.4	*Bifidobacterium adolescentis*	5.8
Sutterella	3.6	*Methylobacterium*	3.2	*Acinetobacter*	3.2	*Bradyrhizobiaceae*	3.5	*Zoogloea*	5.7
Bradyrhizobiaceae	3.6	*Azospira*	3.1	*Prevotella copri*	3.1	*Dorea*	2.7	*Bradyrhizobiaceae*	5.2
Anaerococcus	3.2	*Streptophyta*	2.5	*Pseudomonas fragi*	2.9	*Christensenellaceae*	2.4	*Acidovorax defluvii*	4.5
Roseburia	2.6	*Sedimentibacter*	2.2	*Ruminococcus*	2.9	*Methylobacterium*	2.3	*Azospira*	3.6
Comamonadaceae	2.6	*Carludovica palmata*	2.1	*Bradyrhizobiaceae*	2.8	*Acidovorax defluvii*	2.1	*Prevotella*	3.1
Azospira	2.4	*Halomonas*	2.1	*Prevotella*	2.6	*Rhodovarius lipocyclicus*	1.8	*Enhydrobacter*	2.9
Faecalibacterium prausnitzii	2.0	*MLE1-12*	1.9	*Ruminococcaceae*	2.4	*Sphingomonas*	1.7	*Citrobacter*	1.9
Pseudomonas fragi	1.9	*Bacteroides*	1.5	*Methylobacterium*	2.0	*Veillonella dispar*	1.6	*Comamonadaceae*	1.6
Sphingomonadales	1.7	*Comamonadaceae*	1.4	*Sphingomonadales*	1.9	*Propionibacterium acnes*	1.6	*Methylobacterium*	1.4
Betaproteobacteria	1.5	*Zoogloea*	1.2	*Prevotella stercorea*	1.6	*Anaerococcus*	1.4	*Staphylococcus*	1.1
Bifidobacterium longum	1.5	*Arthrobacter oxydans*	1.2	*Anaerococcus*	1.4	*Acetobacteraceae*	1.2		
Nocardioidaceae	1.3	*Corynebacterium*	1.2	*Rhizobiales*	1.3	*Staphylococcus*	1.1		
Sphingomonas	1.2	*Lachnospiraceae*	1.2	*Comamonadaceae*	1.3	*Rhizobiales*	1.0		
Faecalibacterium	1.0	*Roseburia*	1.1	*Ellin6075*	1.1				
		Lactobacillus iners	1.0	*Corynebacterium*	1.0				
				Rhizobiales	1.0				

C6		C7		C8		C9	
Number of reads:		Number of reads:178416		Number of reads:1666641		Number of reads:205914	
Sequences joined:95,2%		Sequences joined:93,9%		Sequences joined:95,4%		Sequences joined:93,8%	
Methylobacteriaceae	17.0	*Methylobacteriaceae*	23.2	*Cupriavidus*	16.2	*Enterococcus cecorum*	31.1
Cupriavidus	14.0	*Azospira*	14.4	*Methylobacteriaceae*	10.9	*Corynebacterium*	11.5
Bradyrhizobiaceae	7.3	*Bradyrhizobiaceae*	5.6	*Bradyrhizobiaceae*	10.3	*Planococcaceae*	6.7
Lachnospiraceae	5.5	*Cupriavidus*	5.5	*Phaeospirillum*	6.9	*Cloacibacterium*	5.9
Cerasicoccaceae	5.5	*Sutterella*	4.9	*Comamonadaceae*	5.8	*Dokdonella*	3.9
Parabacteroides	4.6	*Streptococcus*	4.6	*Azospira*	4.6	*Sphingomonadales*	3.5
Zoogloea	4.3	*Comamonadaceae*	4.4	*Prevotella copri*	4.4	*Microbacteriaceae*	3.5
Lachnospira	4.1	*Sphingomonadales*	4.2	*Ruminococcaceae*	3.6	*Enhydrobacter*	3.0
Comamonadaceae	3.7	*Sphingomonas*	4.1	*Bacteroides*	3.5	*EW055*	2.8
Acidovorax defluvii	3.3	*Succinivibrio*	4.0	*Streptophyta*	3.3	*Arthrobacter oxydans*	2.7
Bacteroides	3.2	*Acidovorax defluvii*	3.6	*T78*	2.9	*Planctomyces*	2.5
Faecalibacterium prausnitzii	2.9	*Staphylococcus*	2.2	*Veillonella dispar*	2.3	*SR1*	2.4
Prevotella copri	2.6	*Arthrobacter oxydans*	2.0	*Rhizobiales*	2.2	*Ruminococcus*	2.2
Azospira	2.6	*Zoogloea*	1.7	*Zoogloea*	2.2	*Ralstonia*	2.2
Clostridiales	2.2	*Citrobacter*	1.3	*Sphingomonadales*	2.1	*N09*	1.5
Sphingomonadales	1.7	*Corynebacterium*	1.1	*Arthrobacter oxydans*	1.9	*Weeksellaceae*	1.5
Rhodoplanes elegans	1.5	*Propionibacterium acnes*	1.1	*Ralstonia*	1.7	*Prevotella*	1.4
Rhizobiales	1.5			*Bosea genosp*	1.5	*Bifidobacterium adolescentis*	1.2
Streptophyta	1.5			*Corynebacterium*	1.4	*Corynebacterium*	1.1
Comamonadaceae	1.0			*Eubacterium biforme*	1.3	*Rhodospirillaceae*	1.0
				Pseudomonas fragi	1.0		
